# Programming chain-growth copolymerization of DNA hairpin tiles for in-vitro hierarchical supramolecular organization

**DOI:** 10.1038/s41467-019-09004-4

**Published:** 2019-03-01

**Authors:** Honglu Zhang, Yu Wang, Huan Zhang, Xiaoguo Liu, Antony Lee, Qiuling Huang, Fei Wang, Jie Chao, Huajie Liu, Jiang Li, Jiye Shi, Xiaolei Zuo, Lihua Wang, Lianhui Wang, Xiaoyu Cao, Carlos Bustamante, Zhongqun Tian, Chunhai Fan

**Affiliations:** 1 Division of Physical Biology and Bioimaging Center, Shanghai Synchrotron Radiation Facility, Shanghai Institute of Applied Physics, Chinese Academy of Sciences, 201800 Shanghai, China; 2 Department of Chemistry, Institute for Quantitative Biosciences (QB3), Department of Physics, Department of Molecular and Cell Biology, Kavli Energy Nanosciences Institute at Berkeley, Howard Hughes Medical Institute, University of California, Berkeley, CA 94720 USA; 3 State Key Laboratory of Physical Chemistry of Solid Surfaces and College of Chemistry and Chemical Engineering, Xiamen University, 361005 Xiamen, China; 4 School of Chemistry and Chemical Engineering, and Institute of Molecular Medicine, Renji Hospital, School of Medicine, Shanghai Jiao Tong University, 200240 Shanghai, China; 5 Joint Research Center for Precision Medicine, Shanghai Jiao Tong University Affiliated Sixth People’s Hospital South Campus, Fengxian Hospital, 201499 Shanghai, China; 6 Key Laboratory for Organic Electronics & Information Displays and Institute of Advanced Materials (IAM), Jiangsu National Synergistic Innovation Center for Advanced Materials (SICAM), Nanjing University of Posts and Telecommunications (NUPT), Nanjing, 210023 China; 7 Shanghai Key Laboratory of Green Chemistry and Chemical Processes, School of Chemistry and Molecular Engineering, East China Normal University, 200241 Shanghai, China

**Keywords:** Supramolecular polymers, DNA nanostructures

## Abstract

Formation of biological filaments via intracellular supramolecular polymerization of proteins or protein/nucleic acid complexes is under programmable and spatiotemporal control to maintain cellular and genomic integrity. Here we devise a bioinspired, catassembly-like isothermal chain-growth approach to copolymerize DNA hairpin tiles (DHTs) into nanofilaments with desirable composition, chain length and function. By designing metastable DNA hairpins with shape-defining intramolecular hydrogen bonds, we generate two types of DHT monomers for copolymerization with high cooperativity and low dispersity indexes. Quantitative single-molecule dissection methods reveal that catalytic opening of a DHT motif harbouring a toehold triggers successive branch migration, which autonomously propagates to form copolymers with alternate tile units. We find that these shape-defined supramolecular nanostructures become substrates for efficient endocytosis by living mammalian cells in a stiffness-dependent manner. Hence, this catassembly-like in-vitro reconstruction approach provides clues for understanding structure-function relationship of biological filaments under physiological and pathological conditions.

## Abstract (web summary)

Formation of biological filaments via intracellular supramolecular polymerization of proteins occurs under programmable and spatiotemporal control to maintain integrity. Here the authors devise a bioinspired isothermal chain-growth approach to programmably copolymerize DNA hairpin tiles into 1D nanofilaments.

## Introduction

Biological systems have evolved to attain a high degree of organization and adaptability of functions^1,2^. For example, biological filaments (e.g., microtubule filaments and nucleofilaments) that play pivotal roles in organizing cellular structures, in regulating intracellular trafficking, and in maintaining genetic integrity, are dynamically and programmably regulated in vivo^3,4^. Similarly, non-regulated, un-programmed protein polymerization can lead to pathological conditions including many degenerative diseases^5^. Bioinspired in-vitro assembly of biological filaments can be used as experimental platforms to understand their mechanism of generation and to fabricate nano- and micro-devices^6–10^. For example, Aida and coworkers^11^ recently developed a chain-growth mechanism to organize noncovalent interaction-based supramolecular polymerization of single small-molecule monomers. In contrast to the conventional step-growth mechanism, they obtained excellent control over chain length and chirality. Nevertheless, realizing programmable copolymerization from heterogeneous monomers, and/or hierarchical polymerization from supramolecular monomers remain major challenges.

Polymerization of biomolecules (e.g., proteins and nucleic acids) holds great potential for the control of supramolecular organization in vitro. However, with few exceptions^12–14^, structural control of higher-ordered protein assembly in vitro has proven difficult. Unlike proteins, nucleic acids engage in precise and predictable interactions through Watson–Crick base-pairing^15–20^, which have permitted rapid advances in DNA nanotechnology and the construction of prescribed shapes and patterns^21–28^. Thus, pre-designed DNA nanostructures could provide a platform for studying and applying the chain-growth mechanism for programmable living polymerization in vitro and the generation of supramolecular structures.

In this work, we communicate the development of an isothermal chain-growth approach to programmably copolymerize self-assembled DNA hairpin tiles (DHTs) in order to generate hierarchically organized DNA nanostructures. Two types of DHTs assembled from four designed sequences (first-order assembly) are employed as monomers for chain-growth copolymerization (second-order assembly). We demonstrate the formation of shape-defined one-dimensional (1D) DHT nanofilaments and two-dimensional (2D) DHT nanoplatelets (third-order assembly). Finally, we have investigated the cellular uptake of DHT nanofilaments and established a correlation between their stiffness and their endocytic behavior.

## Results

### Programming 1D chain-growth copolymerization of DHTs

**Fig. 1:**
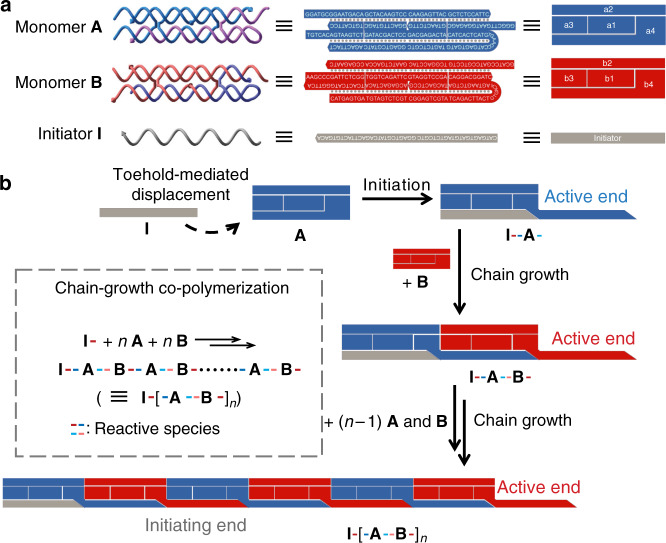
Programmable chain-growth copolymerization of DHTs to form 1D nanofilaments. **a** Schematics of structures (left), sequences (middle), and simplified cartoons of two DHT monomers **A** (blue) and **B** (red) and initiator strand **I** (gray). Each monomer is assembled from four single-stranded DNA. Strand **a1** or **b1** serves as a rigid core linking two crossover junctions. Strands **a2** to **a4** and **b2** to **b4** provide sticky ends for associations with the complementary sequences in the neighboring monomers of the copolymer. Strand **a4** or **b4** also serves as hairpin domain with a toehold to be activated in strand displacement reactions (SDRs) for chain-growth copolymerization. **b** Chain-growth supramolecular copolymerization of monomers **A** and **B** initiated with **I**. The copolymerization starts with the SDR of initiator **I** with monomer **A**. The exposed loop sequence of activated **A** invades the hairpin domain of **B** to activate it with an exposed sequence identical to **I**, which could further activate another monomer **A**. The completed chain-growth copolymerization is achieved by alternating activation and propagation of monomers **A** and **B**. (Inset) Simplified diagram of chain-growth copolymerization

Two basic DHT motifs, **A** and **B**, serve as two metastable monomers for supramolecular copolymerization (Fig. 1a). Each monomer incorporates four pseudoknotted sequences to form a “double-crossover” (DX) motif^18^, in which a pair of crossover junctions holds the two double helices. The DHTs are approximately 2 × 5 × 16 nm in size and explicitly designed to fabricate 1D nanofilaments. Each DHT monomer has three domains: (1) a central core of DNA strand (e.g., strand **a1** or **b1** in Fig. 1a) that links two crossover junctions, and (2) two top corner single-stranded sticky ends (e.g., 5ʹ ends of **a2**, **a4**, **b2**, and **b3** in Fig. 1a), which enable synergetic association with the complementary sequences of neighboring monomers. For example, the 5ʹ sticky end of **a2** is complementary to the 5ʹ end of **b2**, and the 5ʹ sticky end of **a4** is complementary to the 5ʹ end of **b3**. Each monomer also contains (3) a hairpin domain (e.g., **a4** and **b4** in Fig. 1a) that is designed as a long stem and loop sequence (6 nt in length), with the sticky end serving as a toehold (e.g., 5ʹ end of **a3** or 3ʹ end of **b3** in Fig. 1a). The hairpin domain would be opened by a toehold-mediated strand displacement reaction (SDR) and induce a conformational change of the monomer during the subsequent copolymerization^20,29^. Hence, distinct from conventional DX tiles, the DHT monomers can be assembled into periodic patterns via a dynamic chain-growth reaction. Furthermore, a single-stranded initiator (**I**) was designed to match the stem sequence of monomer **A** to trigger the chain-growth copolymerization in a programmable manner.

Chain-growth copolymerization (Fig. 1b, inset) was initiated via the reaction of monomer **A** and initiator **I** that produces a complex carrying a reactive terminus (**I-A**), which then reacted with another monomer **B** to produce an activated complex (**I-A**-**B**). Subsequently, copolymeric chain grew exclusively with sequential addition of monomers **A** and **B** to these active termini. Thus, the chain-growth copolymerization of DHTs was realized via programmable initiation and propagation processes (Fig. 1b and S1). In the absence of initiator strand **I**, the closed loop domains impose a topological constraint and prevent the hybridization between two complementary motifs. Hence, copolymerization of monomers **A** and **B** would not spontaneously proceed at temperatures that did not melt stem domains. Only with the introduction of initiator **I** into the mixture of monomers **A** and **B**, **A** is reactivated via SDR of initiator **I** and the toehold sequence (-TGTCAC-) of **a3**, which leads to the concurrent opening of the hairpin domain of **a4**. The opened hairpin domain exposes the loop sequence of **a4** (-TGAACC-) that serves as an active site for invading the 3ʹ toehold of **b3** (-GGTTCA-) to further activate the hairpin domain of **b4** through another SDR. The exposed loop sequence in **b4** contains an identical segment (-GTGACA-) to the 3ʹ ends of initiator **I**, which further activates monomer **A**. During the chain-growth process, the interactive associations of the top corner sticky ends (5ʹ ends of **a2** with 5ʹ ends of **b2**, 5ʹ ends of **a4** with 5ʹ ends of **b3**) in neighboring monomers also facilitates and stabilizes the copolymer chains. Consequently, once the initiator **I** triggers the SDR with **A**, the subsequent chain-growth occurs in a self-propagating manner to form the supramolecular copolymer with alternate monomers **A** and **B**.

### Characterization, length control, and formation mechanism of 1D DHT nanofilaments

**Fig. 2:**
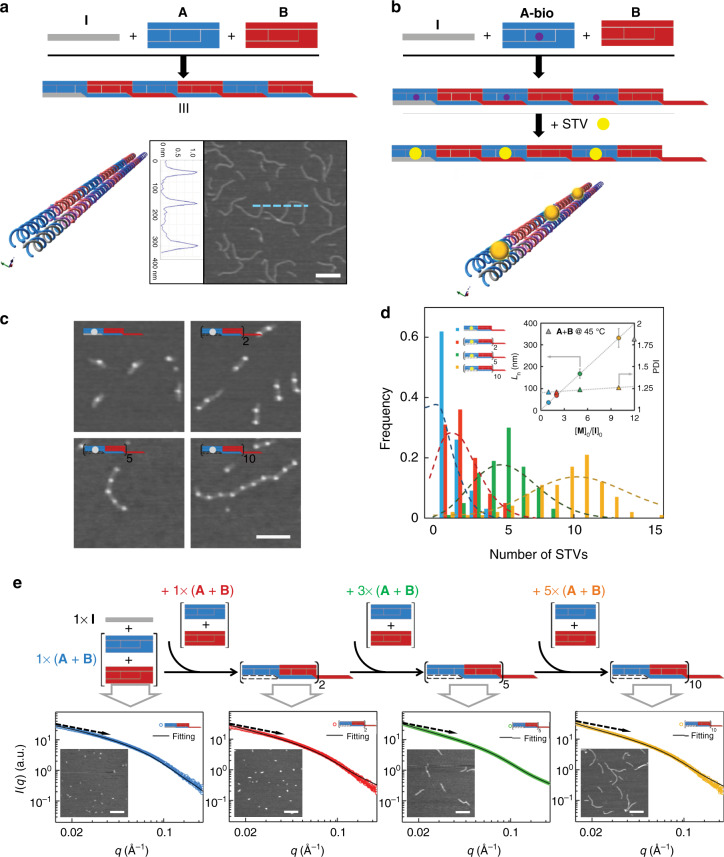
Length control and living 1D chain-growth copolymerization of DHT nanofilaments. **a** Schematic illustration and atomic force microscopy (AFM) image of DHT nanofilaments copolymerized at [**M**]_0_/[**I**]_0 _=  10. Scale bar: 200 nm. **b** Schematic illustrations of the copolymerization of **A-bio**, **B** and initiator **I**, and then labeling with streptavidin (STV) proteins (STVs are shown as yellow dots or balls). **c** AFM images of the STV-decorated DHT nanofilaments copolymerized at the ratios of [**M**]_0_/[**I**]_0_ = 1, 2, 5, and 10, respectively. Scale bar: 100 nm. **d** Histogram of the number of STVs that decorated the DHT nanofilaments copolymerized at individual ratios of [**M**]_0_/[**I**]_0_ = 1 (blue), 2 (red), 5 (green), and 10 (yellow). The dashed lines show Poisson fits to the histograms with the corresponding colors. Inset shows plots of average lengths (*L*_n_, left) and PDI (right) vs. [**M**]_0_/[**I**]_0_ ratios for DHT nanofilaments and PDI of copolymer formed at 45 °C without initiator (gray triangle). Error bars represent the mean ± standard deviation. **e** Schematic illustration, small-angle X-ray scattering (SAXS) profiles and AFM images (inset) of living chain-growth copolymerization with the stepwise addition of new monomers into the polymerization system. Scale bar: 200 nm

DHT monomers **A** and **B** were pre-assembled with equivalents of four sequences (**a1** to **a4** and **b1** to **b4**. Supplementary Table 1 lists the sequences of all the oligonucleotides used in constructing DHT monomers), separately. Native polyacrylamide gel electrophoresis (PAGE) analysis revealed that the DHT monomers were assembled with high efficiency (Fig. 2). The chain-growth copolymerization was carried out in an aqueous solution containing 1 μM equivalents of monomers **A** and **B** and 0.1 μM initiator **I** ([**A**]_0_/[**I**] _0_ = [**B**]_0_/[**I**]_0_ = [**M**]_0_/[**I**]_0 _= 10). The mixture solution was incubated at room temperature (r.t.) for 1 h, during which monomers sequentially propagated into a supramolecular copolymer. As illustrated in atomic force microscopy (AFM) images (Fig. 2a and Supplementary Figure 3), the linear structures of DHT nanofilaments were formed via copolymerization of two DHT monomers with a polydispersity index (PDI) range of 1.21–1.26 (PDIs, Fig. 2d, inset and Supplementary Table 2)^30^, which is much lower than that of polymers formed upon heating without initiator strand (Fig. 2d, inset, Supplementary Figure 4). AFM profile analysis further revealed that the DHT nanofilaments have a near-uniform height of 1.2 nm (Fig. 2a, inset). No polymerization happens in the absence of initiator strand (Supplementary Figure 5).

Supramolecular polymerization often proceeds through step-growth mechanisms to assemble monomers into a plethora of functional polymers^31–33^, which are nevertheless generally of high dispersity. Importantly, living polymerization, as a type of chain-growth polymerization, provides an efficient approach to achieve precision and control over macromolecular organization. Specifically, formation of nanofilaments of low dispersity is regulated through the control of the polymerization processes: the monomers undergo assembly only upon reacting with an initiator molecule to create an active site, which is continuously regenerated upon the binding of a new monomer. The exhaustion of monomers terminates the growth whereas the addition of new monomers resumes it. Hence, fine control of the concentration of monomer can in principle result in low dispersity. Accordingly, we tuned the molar ratios of monomers **A** and **B** with initiator **I** ([**M**]_0_/[**I**]_0_) from 1, 2, 5, to 10, whereas the concentration of monomers was kept constant ([**A**]_0_ = [**B**]_0_ = [**M**]_0 _= 1 μM). Moreover, to directly visualize the structure of copolymeric DNA nanofilaments, we utilized biotin-specific streptavidin (STV) as a label for AFM imaging. STV was site-specifically anchored at the center of monomer **A**, which has a biotin molecule at the 5ʹ end of core strand **a1** (Fig. 2b, monomer **A-bio**). Using AFM imaging, we observed the presence of different numbers of STVs as bright dots located in the prescribed positions of monomer **A** in DHT nanofilaments (Fig. 2c). The discrete patterns of anchored STVs allow direct visualization of copolymeric assemblies with nanometer resolution at the single-molecular level. The histogram shown in Fig. 2d represents the length distribution of DHT nanofilaments obtained by counting the number of STV-decorated chains copolymerized at specific monomer-to-initiator ratios ([**M**]_0_/[**I**]_0_) (Supplementary Figure 6 and 7). Specially, when the monomers and initiator were in an equivalent concentration ([**M**]_0_/[**I**]_0 _=  1), 62% of the copolymeric DHT nanofilaments were decorated with single STV, as expected from the stoichiometry of the **I-A-B** complex. The average contour length (*L*_n_) of the assembled DNA nanofilaments increases linearly with the [**M**]_0_/[**I**]_0_ ratio (Fig. 2d). The histograms for the number of decorated STVs can be well fitted using the Poisson distribution^34^, characteristic of living polymerization.

To substantiate the living nature of the supramolecular copolymerization of DHT monomers, we carried out an experiment by stepwise adding monomers to the polymerization system. New monomers were added to the pre-formed copolymer solution ([**M**]/[**I**] ratio = 1:1) and incubated at r.t. Each copolymeric nanofilament with low dispersity was characterized with small-angle X-ray scattering (SAXS), AFM and dynamic light scattering (DLS) measurements (Fig. 2e and Supplementary Figure 8). The SAXS profiles showed that the length of the copolymer increased continuously with the addition of new monomers (Supplementary Table 2). Both the contour length measure from AFM and hydrodynamic diameter from DLS confirmed this trend. Hence, the copolymeric nanofilaments retain active sites for continuous polymerization with the supply of new monomers, which is characteristic of living supramolecular polymerization.

**Fig. 3:**
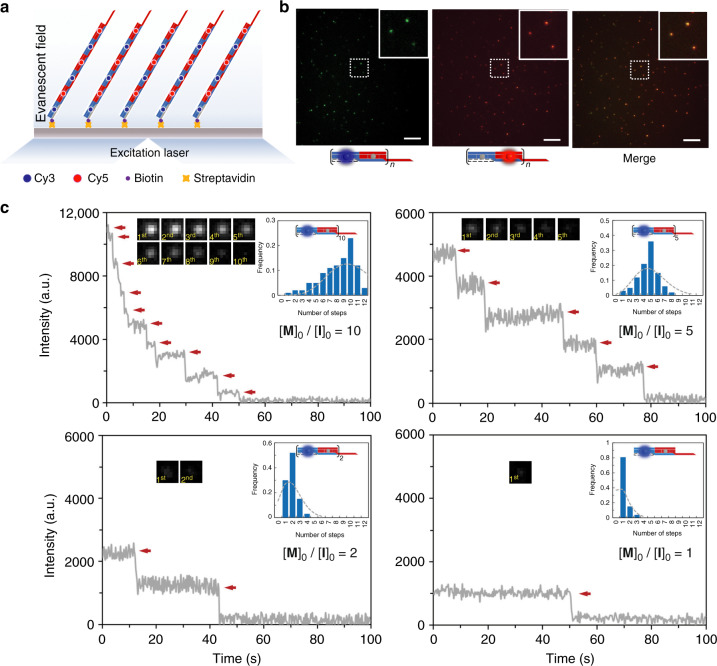
Single-molecule fluorescence imaging of copolymerization of 1D DH nanofilaments. **a** Schematic illustration of the fluorophore-labeled nanofilaments (copolymerized at the ratio [**M**]_0_/[**I**]_0_ = 2) imaged through total internal reflection fluorescence microscopy (TIRFM). Cy3 (blue dot) and Cy5 (red dot) are labeled in the core strands **a1** and **b1**, respectively. **b** TIRFM images of surface-grafted nanofilaments acquired with the excitation of 561 nm (left) and 633 nm (middle). Overlay image (right) shows the colocalization of Cy3 (green) and Cy5 (red) labels. **c** Representative intensity vs time trajectories illustrating the fluorescence photobleaching of Cy3-labeled nanofilaments copolymerized with ratios of [**M**]_0_/[**I**]_0_ = 10, 5, 2, and 1, respectively (arrows indicate photobleaching events)

Total internal reflection fluorescence microscopy (TIRFM) allows quantitative single-molecule analysis of the copolymeric DHT nanofilaments. Fluorescent dyes Cy3 and Cy5 were attached to the 5ʹ ends of **a1** and **b1**, becoming located at the centers of the **A** and **B** monomers, respectively (Fig. 3a and S9). The initiator **I** was labeled with a biotin moiety at the 3ʹ end, allowing it to be immobilized onto a STV-decorated glass surface via biotin–STV interaction. The chain-growth copolymerization of Cy3-labeled **A**, Cy5-labeled **B**, and biotin-labeled **I** proceeded in solution for 1 h at r.t. The assembled DHT nanofilaments were then incubated with STV-decorated glass slides and characterized using TIRFM via independent recording of Cy3 (red) and Cy5 (green) emissions. Overlay analysis of the images obtained from each channel revealed the colocalization of Cy3 and Cy5 emissions, indicating the integrity of the assembled DNA structures (Fig. 3b), and the absence of nonspecific aggregation.

Quantitative analysis of DHT nanofilaments was further performed by using a single-molecule fluorescence photobleaching protocol^35^. Cy3 on **A** was excited in an evanescent field by a continuous-wave diode-laser with a 561-nm emission, and photobleaching events were recorded simultaneously. As shown in Fig. 3c, the fluorescence intensity of each individual copolymeric DHT nanofilaments decreased stepwise upon excitation, owing to sequential bleaching of labeled Cy3. By counting the number of discrete intensity levels in the resulting “staircase” patterns, we determined the number of DHT monomers in each individual nanofilament. Samples assembled from different ratios of [**M**]_0_/[**I**]_0_ (10, 5, 2, and 1) were analyzed with this photobleaching method. As shown in Fig. 3c, the fluorescence intensity of nanofilaments copolymerized under different [**M**]_0_/[**I**]_0_ ratios decreased in a stepwise manner over time with the total number of steps decreasing in the proportions of 10, 5, 2, and 1, in accordance with the expected number of Cy3 on the corresponding nanofilaments. Each image was recorded at a time point of each intensity step. The distribution histogram for the number of Cy3, obtained by counting the number of photobleaching events recorded for each of the 100 nanofilaments, revealed that 23, 36, 52, and 81% of the nanofilaments exhibited 10, 5, 2, and 1 intensity steps, respectively (Fig. 3c, inset), which further confirmed the formation of copolymeric structures of **I**-[**A-B**]_*n*_ (*n* = 10, 5, 2, and 1).

### Programming 2D chain-growth copolymerization of DHTs

**Fig. 4:**
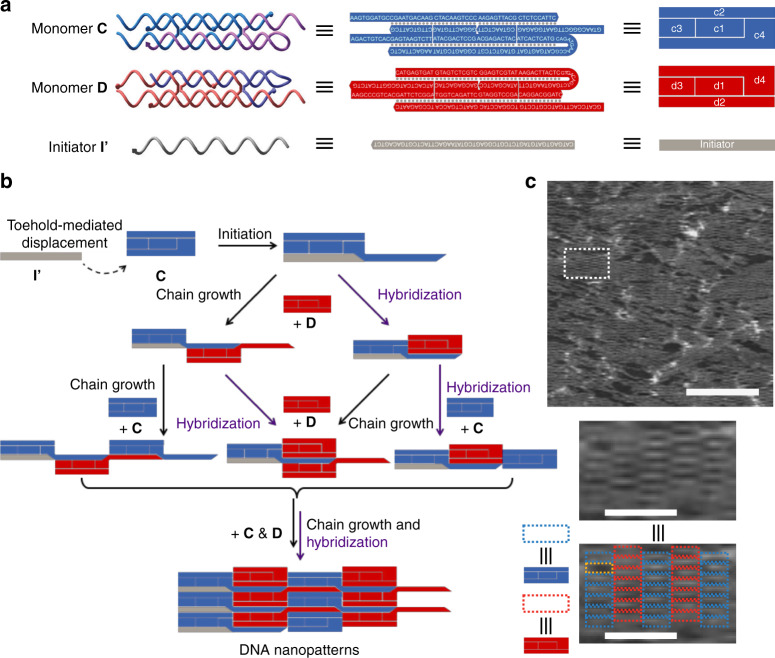
Programmable 2D chain-growth copolymerization of DHT nanoplatelets. **a** Schematics of structures (left), sequences (middle), and simplified cartoons of two monomers **C** (blue) and **D** (pink) and initiator strand (**I’**). **b** 2D chain-growth supramolecular copolymerization of monomers **C** and **D** initiated with **I’**. Copolymerization starts with the strand displacement reaction of initiator **I’** with monomer **c3**, exposing the loop sequence of **c4** to further invade the hairpin domain of **D** and open the **d3**. The opened **d3** contains an identical toehold to **I’**, which can further activate the monomer **C**. Meanwhile, supramolecules are assembled with the associations of monomers’ corner sticky ends in a direction perpendicular to the helical axis. **c** Atomic force microscopy (AFM) images of 2D DHT nanoplatelets. Scale bars: 200 nm. (Inset) Zoomed-in AFM image. Dashed line squares indicate monomers **C** (blue) and **D** (red). Scale bars: 50 nm

Having demonstrated filament generation by chain-growth polymerization, we reasoned that DNA self-assembly and chain-growth copolymerization could be used to increase the hierarchical complexity of assembled supramolecular structures into higher dimensions. Figure 4 depicts the strategy utilized to obtain 2D chain-growth copolymerization. The designed DHT monomers **C** and **D** and initiator **I’** for 2D copolymerization are shown in Fig. 4a (Supplementary Table 3 lists the sequences of all the oligonucleotides used in constructing DHT monomers). Here, the DHTs are approximately 2 × 5 × 18 nm in size, which are longer than the monomers used for 1D copolymerization. Due to the increased length of the strands, the 5ʹ sticky ends of **c2** are complementary to the 5ʹ ends of **d2**, whereas the 5ʹ ends of **c4** binds the 5ʹ ends of **d3** (Fig. 4b). After being activated by initiator **I’** in a toehold-mediated displacement reaction, monomer **C** with an exposed hairpin strand can invade monomer **D**. The separation between the two intermolecular crossover junctions was designed to be 32 nt, or about 3 helical turns, compared with the separation of 26 nt (about 2.5 helical turns) present in the monomers used for the generation of 1D copolymers. Thus, monomer **D** binds on the opposite side of chain-growth strands (Fig. 4b). With the synergistic combination of chain-growth copolymerization (parallel to the DNA helical axis) and DNA overhangs associations (perpendicular to the DNA helical axis), DHT monomers were assembled in two dimensions to form hierarchical nanoplatelets (Supplementary Figure 10). Accordingly, AFM images showed distinguishable interlaced monomers patterned in two dimensions (Fig. 4c).

### Endocytosis of DHT nanofilaments in living cells

**Fig. 5:**
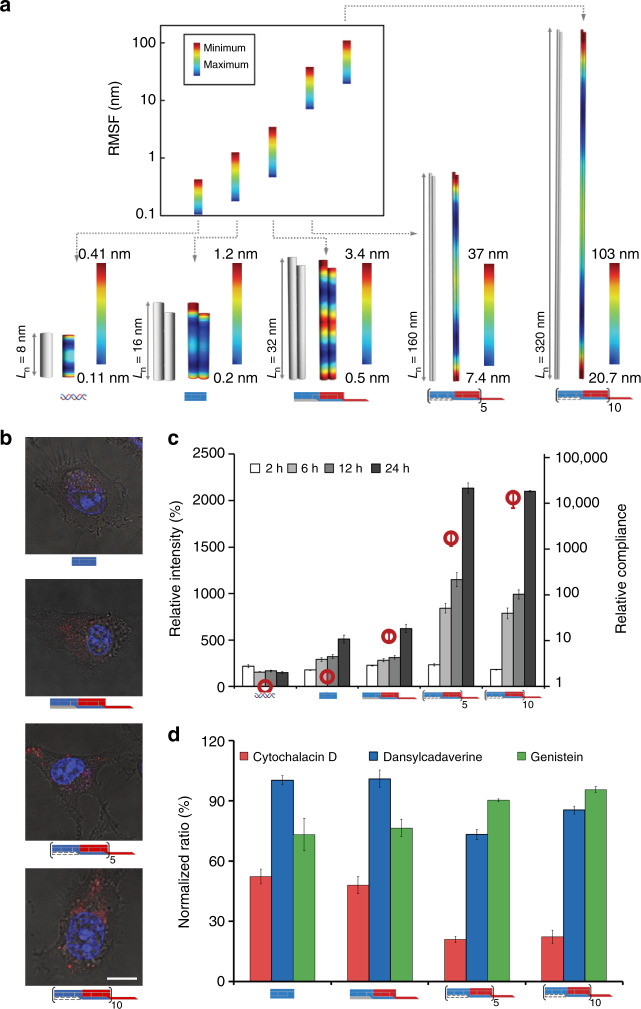
Endocytosis of 1D DHT nanofilaments in living mammalian cells. **a** Three-dimensional (3D) equilibrium conformation and heat map color range for root-mean-square fluctuations (RMSFs) simulated by CanDo. Blue and red color represent low and high relative flexibility, respectively. Bluest color indicates zero percentile RMSF and reddest color indicates no <95% RMSF. Insets shows plots and table of minimum and maximum RMSF values of double-stranded DNA (dsDNA), monomer **A** and the nanofilaments of different lengths (i.e., **I**-**A**-**B**, **I**-[**A**-**B**]_5_, and **I**-[**A**-**B**]_10_). **b** Merged confocal images of intracellular localization of Cy3-labeled DNA structures (red) under serum-free incubation condition for 12 h, with cell nuclei stained with SYTO 59 (blue). Scale bars: 10 μm. **c** Analysis of the dependence of cellular uptake on the structural properties and time. DNA materials (the concentration was normalized as 50 nM of labeled dyes in dsDNA, monomer **A**, **I-A-B**, **I**-[**A**-**B**]_5_, and **I**-[**A**-**B**]_10_, respectively) were incubated with A549 cells at different time intervals in the serum-free medium. Fluorescence was quantified with flow cytometry. The results are representative of three independent experiments. **d** Fluorescence analysis of endocytic mechanisms of DHT and DHT nanofilaments. Different endocytosis inhibitors were applied to elucidate the associated mechanisms. All error bars represent the mean ± standard deviation obtained from at least three independent experiments

Nucleic acid-based nanostructures have been extensively explored in diagnostic and therapeutic applications owing to their excellent inherent biocompatibility and precisely predefined shapes.^36–39^ Previous studies have revealed the dependence of cellular uptake properties on the aspect ratio of various materials^40^. Given that chain-growth DNA copolymerization provides a generic means to construct DNA nanostructures with controllable aspect ratios and high chemical/biochemical stability (Fig. 5a, Supplementary Figures 11 and 12), we next characterized the endocytosis of differently sized DHT nanofilaments.

To this end, we compared the endocytosis of double-stranded DNA (dsDNA) of 24-bp (8.2 nm), the monomer **A** and copolymerized DHT nanofilaments of different lengths (i.e., **I-A-B**, **I-**[**A-B**]_5_ and **I**-[**A-B**]_10_) by A549 mammalian cells (Fig. 5a). These DNA materials were labeled with Cy3, which could be quantified using confocal microscopy and flow cytometry (Figs. 5b, c and Supplementary Figure 13). Upon incubation of the DHT nanofilaments with the A549 cells for different times in serum-free conditions, flow cytometry revealed that the fluorescence intensity per cell correlated strongly with the aspect ratio of the DNA materials (Fig. 5c and Supplementary Figure 14). Specifically, copolymerized nanostructures with higher aspect ratio (**I**-[**A-B**]_5_ or **I**-[**A-B**]_10_) showed remarkably higher cellular uptake than those with lower ones, i.e., dsDNA, DHT (monomer **A**), and **I-A-B**. Importantly, the fluorescence intensity for DHT and DHT nanofilaments (*i.e.*, monomer **A**, **I-A-B**, **I**-[**A-B**]_5_, and **I**-[**A-B**]_10_) increased intracellularly in a time-dependent manner. Although dsDNA showed uptake to some extent, its fluorescence did not exhibit time-dependent increase, suggesting that the observed fluorescence primarily originated from surface-adsorbed dsDNA rather than from internalized species. Further characterization by confocal microscopy confirmed that the Cy3-labeled DHT nanofilaments (red) were located largely inside the cytosol (Fig. 5b). The fluorescence of **I**-[**A-B**]_5_ or **I**-[**A-B**]_10_ was much more intense than that of **A** or **I-A-B**, a result consistent with the flow cytometry results. Furthermore, we tested the dependence of internalization on other two cell lines (Hela and MCF-7), the results illustrated the similar increasing trends of uptake efficiency along with the shape and complicance of DNA structures (Supplementary Figure 15).

Having established the importance of nanofilament formation for effective cellular uptake, we further explored the quantitative relationship between the stiffness of DHT nanofilaments with their uptake efficiency. The bending stiffness of a beam increases with its effective radius, but decreases inversely proportional to its length. By treating these DNA materials as beam-shaped structures, we calculated their relative mechanical compliance (defined here as the ratio of bending stiffness of dsDNA, with a length of 24 bp, to the bending stiffness of given DHT nanofilaments, *k*_b_ (dsDNA)/*k*_b_ (nanofilament), see also Supplementary Figure 18 and Supplementary Table 4)^41,42^ and simulated their structural conformation and mechanical compliance using program CanDo^43,44^ (Fig. 5a). The two ends of DHT monomer and nanofilaments of different lengths are floppy and exhibit largest degree of compliance (root-mean-square fluctuation (RMSF) red color), which are corresponding to the flexible stick ends and loop domains. The middle portion of nanofilaments show relative high fluctuations (RMSF yellow or red color). The inset figure exhibit the comparison of relative RMSF of dsDNA, monomer **A** and nanofilaments. In all, 24-bp dsDNA and monomer **A** show lowest flexibility and copolymeric nanofilaments show relative higher compliance. It exhibits the increasing trend of compliance along with the [**M**]/[**I**] ratios. Importantly, DHT nanofilaments with higher relative compliance showed more efficient cellular uptake (Fig. 5c). Given that the majority of exhibited fluorescence for dsDNA came from surface adsorption, its cellular uptake is regarded to be only marginal. Hence, the formation of well defined yet flexible DHT structures appears to be a prerequisite for efficient cellular uptake when compared with less flexible 24-bp dsDNA. We also performed DLS measurements of DNA nanofilaments after incubation in the cell culture medium (FBS-free 1640, Supplementary Figure 11). Compare with DLS performed in the common buffer (1 × TAE/Mg^2+^ buffer, Supplementary Figure 8), the trend that the average hydrodynamic diameters of the products increased along with the [**M**]/[**I**] ratios, remains the same. However, DLS in the culture medium exhibited narrower waves, with the peaks shifted to the lower hydrodynamic diameter range, especially for the copolymer formed at [**M**]/[**I**] = 10:1, which suggest that the nanofilaments are more compact in the cell culture medium. Hence, both the compactness and the stiffness play important roles in cell uptake, which might have important biological implications.

To further explore the mechanism of DHT nanofilament endocytosis, cells and dye-labeled DNA structures were incubated at 4 °C, wherein the energy-dependent endocytosis is unfavorable. Flow cytometry results indicated that the cellular uptake of all DHT nanofilaments showed remarkable decrease (68–78%) (Supplementary Figure 16), supporting the conclusion that DHT nanofilaments were actively internalized via energy-dependent endocytosis. In contrast, dsDNA signals showed minimal decrease upon temperature change, which further confirmed that its endocytosis was negligible. The endocytic pathway of the DHT nanofilaments was additionally investigated by using selective pharmacological inhibition. Before the uptake experiments, cells were treated with a series of inhibitors (Supplementary Table 5) that can respectively inhibit pinocytosis (cytochalasin D), clathrin-mediated endocytosis (dansylcadaverine), and caveolae-mediated endocytosis (genistein). Uptake of all nanostructures showed significant decrease by cells treated with cytochalasin D; specifically, the uptakes were reduced by 48, 52, 79, and 78% relative to those of the untreated cells for **A**, **I-A-B**, **I-**[**A-B**]_5_, and **I**-[**A-B**]_10_, respectively (Fig. 5d). In contrast, the other two inhibitors, dansylcadaverine and genistein, did not induce significant reduction of cellular uptake. These results indicate the two DHT nanofilaments, **I**-[**A-B**]_5_ and **I**-[**A-B**]_10_, were internalized mainly via the macropinocytosis pathway, whereas **A** and **I-A-B** might take multiple pathways. The use of another macropinocytosis inhibitor, nocodazole, also led to an inhibition effect similar to cytochalasin D, confirming that DHT nanofilaments take the macropinocytosis pathway (Supplementary Figure 17). It is worthwhile to point out that macropinocytosis is an endocytic pathway related to cell surface ruffling, which provides a route for non-selective endocytosis of soluble particles with sizes larger than 150–200 nm^45^, which matches well the length scale of DHT nanofilaments (170 ± 21 nm and 332 ± 45 nm).

## Discussion

In this work, we have demonstrated the supramolecular organization of DNA nanostructures via programmable chain-growth copolymerization of self-assembled DHT monomers. Unlike DNA origami that has high design precision, it is often difficult to employ tiles to construct DNA shapes with prescribed size and geometry^16,18,26,46^. The dynamic assembly of DNA tiles thus represents a conceptual advance in structural DNA nanotechnology, which extend the conventional assembly of static DNA nanostructures to more active assembly of hierarchical nanostructures.

This “catassembly”-like^47^ chain-growth copolymerization method provides a generic approach for spatiotemporal control of biomolecular structures in test tubes and potentially in living cells, which should be readily adaptable for assembling versatile types of DNA motifs, e.g., origami^16,23^ and single-stranded tiles^26^. Chain-growth copolymerization represents a distinct mechanism in supramolecular assembly of low-dispersity polymers, which was previously realized only in organic solvents^11^. The work presented here demonstrates chain-growth copolymerization of DNA tiles in aqueous solution, which compares favorably with previously reported DNA hybridization chain reactions with leads, nonetheless, to high product dispersity^48–51^. This synthetic approach, along with the use of single-molecule imaging techniques^52^, open doors for characterizing the mechanisms of living supramolecular organization in vitro and in vivo.

Furthermore, we establish that the formation of flexible DNA nanofilaments is a critical factor for their effective cellular uptake, which depends both on their geometric and mechanical properties. Moreover, the strong dependence of cellular uptake with the size and shape of DNA nanostructures provides important clues for the use of these assemblies in diagnostic and therapeutic applications, including drug delivery and smart nanorobots^37,53–55^.

## Methods

### Materials

The sequences of DNA oligonucleotides were constructed in silico by using Seeman’s program SEQUIN^56^ with the principle of sequence symmetry minimization algorithm^57^. DNA sequences used in the experiment were shown in Fig. 1 and Supplementary Tables 1 and 2.

All DNA strands were synthesized and purified by Invitrogen Life Technologies (Shanghai, China) and used without further purification. The concentration of each strand was estimated by measuring the UV absorbance at 260 nm using a Hitachi U-3010 spectrophotometer (Hitachi, Japan). DNA oligonucleotides labeled with biotin, Cy3, or Cy5 were purified by High Performance Liquid Chromatography (HPLC), whereas other oligonucleotides were purified by PAGE by Invitrogen. STV molecules were purchased from Sigma-Aldrich Co. LLC. (United States) and used without further purification.

The dsDNA was synthesized by mixing two completely complementary oligonucleotides (strands a3 and coa3) in 1 × TAE/Mg^2+^ buffer with a further incubation for 2 h at r.t.

### Self-assembly of DHT monomers

The oligonucleotides of each DHT monomer were stoichiometrically mixed in a 1 × TAE/Mg^2+^ buffer containing 40 mM Tris base (pH 8.0), 20 mM acetic acid, 2 mM EDTA, and 12.5 mM magnesium acetate. Then, the DNA strands solution was slowly cooled down from 95 °C to r.t. over 24 h in water bath insulated in a styrofoam box.

### Non-denaturing PAGE

In all, 10 μL of each annealed sample was subjected to the 8% PAGE (19:1 acrylamide/bisacrylamide in 1 × TAE/Mg^2+^ buffer). Gels were run at 100 V (constant voltage) for about 2 h with electrophoresis apparatus (Bio-Rad, United States). Then, the gels were stained with 1 × GelRed nucleic acid dye (Biotium, United States) and scanned under UV light.

### 1D chain-growth copolymerization of DHTs

To copolymerize the 1D nanofilaments, 1 μM of monomers **A** and **B** at equimolar concentrations was mixed with initiator strand **I** in different ratios, from 1:1, 1:0.5, 1:0.2 to 1:0.1. The mixtures were incubated in r.t. for 1 h and then were characterized by AFM and TIRFM (see Fig. 3 and Supplementary Figure 3).

To demonstrate the living nature of DHT copolymerization, we carried out the experiments by mixing 1 μM of monomers **A** and **B** at equimolar concentrations with initiator strand **I** ([**M**]/[**I**] = 1:1), which were then incubated at r.t. for 1 h. Then, we stepwisely added more monomers **A** and **B** into the pre-formed copolymers at varied final monomer/initiator molar ([**M**]/[**I**]) ratios of 2:1, 5:1, and 10:1, respectively, followed by further incubation. After each monomer addition and incubation, the mixture was characterized using SAXS, AFM, and DLS measurements (see Fig. 2e and Supplementary Figure 8).

### Thermal copolymerization of DHTs without initiator

Monomers **A** and **B** at equimolar concentrations (1 μM) were mixed and heated at 45 °C for 1 h and then incubated at r.t. for 30 min. The products from copolymerization of monomers without initiators were characterized with AFM (see Supplementary Figure 4).

### 2D chain-growth copolymerization of DHTs

To copolymerize the 2D nanoplatelets, 1 μM of monomers **C** and **D** at equimolar concentrations were mixed with initiator strand **I’** in the molar ratio of 1:10. The mixture was incubated in 37 °C for 4 h and then was characterized by AFM (see Fig. 4c).

### Biotin–STV binding assays

The strand a1 was synthesized with biotinylation at 5ʹ end. After biotin-labeled DHT nanofilaments were constructed as described, a stoichiometric amount of STV in 1 × TAE/Mg^2+^ buffer was added and the finial molar ratio of STV to biotin nanofilament was 10:1. The mixture was left at r.t. for 5 min and then was characterized by AFM (see Fig. 2c and Supplementary Figure 6 and 7).

### AFM characterization and analysis

Each sample of 2–3 μL was deposited on a freshly cleaved mica surface and was left to adsorb on the surface for 3 min. For AFM imaging in air, the mica surface was slowly rinsed with water for three times (each time with 100 μL water) to remove the salt. Then, the mica surface was dried with a mild air stream by an ear-washing bulb and was imaged with a MultiMode 8 AFM with NanoScope V Controller (Bruker, Inc.) under tapping mode in air. For AFM imaging in fluid, 30 μL of 1 × TAE/Mg^2+^ buffer was added to cantilever holder and then the mica surface with sample buffer was imaged with AFM using tapping mode in fluid. All AFM images were analyzed by NanoScope Analysis v1.50.

### Statistical length and decorated STVs numbers of DHT nanofilaments analysis

The contour lengths and decorated STVs numbers of DHT nanofilaments were estimated from the AFM images manually using the ImageJ software (National Institutes of Health, Bethesda, MD, http://imagej.nih.gov/ij/). For the statistical analyses, 100 objects of each nanofilament copolymerized at different [**M**]_0_/[**I**]_0_ ratios were processed to estimate the contour lengths and STVs numbers. Histograms were constructed and values of the number-averaged length (*L*_n_), weight-averaged length (*L*_w_), standard deviation *σ*, and PDI^58^ (see Supplementary Table 2) were estimated using the following equations where *N* is the number of objects for each sample:

1 $${L}_{\mathrm{n}} = \frac{{\mathop {\sum }\nolimits_{i = 1}^n N_iL_i}}{{\mathop {\sum }\nolimits_{i = 1}^n N_i}}$$

2 $${L}_{\mathrm{w}} = \frac{{\mathop {\sum }\nolimits_{i = 1}^n N_i{L_i}^2}}{{\mathop {\sum }\nolimits_{i = 1}^n N_iL_i}}$$

3 $$\sigma = \sqrt {\frac{1}{N}\mathop {\sum }\nolimits_{i = 1}^n \left( {x_i - \mu } \right)^2}$$

4 $${\mathrm{PDI}} = \frac{{L_{\mathrm{w}}}}{{L_{\mathrm{n}}}}$$

### DLS measurements

DLS experiments were carried out on a Zetasizer, Nano Series, Nano ZS machine (Malvern Instruments). The software Dispersion Technology Software 5.10., from Malvern Instruments was used for data analysis. All measurements were repeated for three times.

### SAXS measurements

SAXS experiments were carried out at the beamline BL19U2 of the National Center for Protein Science Shanghai (NCPSS) at Shanghai Synchrotron Radiation Facility (SSRF). The wavelength (*λ*) of the X-ray was set at 1.023 Å. Scattered X-ray intensities were measured by using a Pilatus 1 M detector (DECTRIS Ltd). The sample-to-detector distance was 2650.0 mm for these measurements, it was set such that the detecting range of momentum transfer *q* (*q* = 4πsin(*θ*)/*λ*, where 2*θ* is the scattering angle) of the SAXS experiments was 0.01–0.5 Å^−1^. The solution temperature was held constant at r.t.

A flow cell comprising a cylindrical quartz capillary with a diameter of 1.5 mm and a wall thickness of 10 μm was used, and the exposure time was set at 1 s. The X-ray beam, with a size of 0.40 × 0.15 (H × V) mm^2^, was adjusted to pass through the centers of the capillaries for each measurement. In all, 100 µL of each prepared sample was used for SAXS experiment. To obtain optimized signal-to-noise ratios, 20 frames were recorded for each sample.

The 2D scattering images were converted to 1D SAXS curves through azimuthal averaging after solid angle correction and then normalizing to the intensity of transmitted X-ray beam using the software package BioXTAS RAW 1.2.1.^59^ The data were further analyzed by the software SasView^60^.

### TIRFM

The slides and coverslips were rinsed with deionized MilliQ water (resistivity of 18.5 M·Ω). In order to remove other fluorescent and nonspecific impurities adsorption on the slides surfaces, the slides were immersed in base piranha solution (5% ammonium hydroxide, 14% hydrogen peroxide) for 30 min and sonicated in 1 M KOH for another 30 min. The slides were then rinsed with deionized water and left in dried state for further use. The piranha etching makes the slides surface hydrophilic by generating hydroxyl groups. For amino silanization, the clean slides were immersed into a 5% 3-aminopropyltriethoxysilane solution with acetone for 1 h, followed by acetone rinsing cycles and drying for 1 h at 80 °C. To decorate the STVs onto the surface, the slides were incubated in a mixture solution of 10% pyridine and 0.2% para-phenylene diisothiocyanate (PDITC) in *N*, *N*-dimethylformamide (spectroscopic grade) for 2 h. The amino silanized surface of slides was then covalently coated with a layer of the bifunctional crosslinking agent PDITC. The slides were rinsed thoroughly with methanol and acetone, then about 100 µL of 0.5 mg/mL STV was applied to each slide of the slides. Coverslips were placed over each slide to spread solution evenly and to prevent drying. The coverslip and slide sandwiches were incubated in a humid and closed container at r.t. for 2 h. The coverslips were then removed and the slides were rinsed thoroughly in turn with deionized MilliQ water, 1 M NaCl, 40 mM NaOH and deionized MilliQ water again. The STV-coated slides were dried under nitrogen and stored at −20 °C for use.

The imaging chambers were constructed by using a pair of STV-coated slide and a coverslip. A slide on a flat surface with the STV-coated side was placed facing up and then double-sided sticky tapes were put over the slide to create a channel on the STV-coated surface. Notice that the predrilled holes were positioned at the center of the channel and were not blocked. A coverslip was placed on top to complete the chamber and press the coverslip over the sticky tapes placed area. The chambers were constructed with double-sided sticky tape as a spacer. Samples with biotinylated DNA were introduced through the hole in the slide for single-molecule fluorescence imaging.

The microscope used was a Leica AM True MultiColor Laser TIRF. Lasers with the 561 nm and the 635 nm were employed to independently excite the Cy3 and Cy5 dyes, respectively. The samples were excited with an exposure time of 210 ms. The fluorescence emission was collected using an N.A. = 1.47, 100 × oil immersion objective and images were captured by an EMCCD camera (Hamamatsu-C9100–13–360237) with a frame rate of 3.88 Hz and the view size of 81.87 × 81.87 µm^2^. Fluorescence emission was chromatically separated using 11523015/BZ00 fluorescent filter cube (filters: BP610/65 and BP720/60; dochroic mirrors: 550 and 650, LEICA). The intensity-time traces were obtained by region-of-interest (ROI) analysis of a video with a build-in program of the microscope’s control software. The number of steps in intensity-time traces were evaluated and counted manually.

In this experiment, 1 μM of monomers **A** and **B** at equimolar concentrations was mixed with the initiator strand (with ratios from 1:0.1, 1:0.2, 1:0.5 to 1:1) in solution. The mixtures were then incubated at r.t. for 1 h. The prepared samples were diluted to 50 pM for incubation with STV-coated glass for 5 min, which were then ready for TIRF measurements.

### Cellular uptake assay with flow cytometry

A549 cells were grown in PRIM-1640 (Invitrogen, USA) with 10% heat-inactivated fetal bovine serum and antibiotics (100 µg/mL of streptomycin and 100 U/mL of penicillin) at 37 °C with 5% CO_2_. Cells were seeded in 24-well plates and grown overnight prior to studies. Cells were washed two times with phosphate-buffered saline (PBS, pH = 7.4), incubated with dye-labeled DNA nanostructures (the finial concentration was estimated as 50 nM of labeled dyes for each structure) in cell culture medium (500 μL), containing FBS [10% (vol/vol)] or not at 37 °C for different times (2, 6,12, 24 h), followed by washing with PBS three times. Then, cells were digested with 0.25% trypsin (Gibco, USA) and resuspended in PBS (200 μL). Cells with the same procedure but without DNA nanostructures were used as controls. Samples of at least 5000 cells were analyzed in triplicate using flow cytometry (FACSArray; BD Biosciences, San Jose, CA, USA). Consistent gating based on cell size and granularity (forward and side scatter) was applied to select only fluorescence measurements from healthy cells and exclude cell debris and doublets. Gating region indicates the main population of the cells we analyzed (Supplementary Figure 19).

### Confocal microscopic imaging

A549 cells (~5 × 10^4^) were seeded in four-chamber glass bottom confocal dish (35 mm with 20 mm bottom well, In Vitro Scientific, USA) and grown overnight prior to studies. Then, cells were washed two times with PBS (pH = 7.4), incubated with fluorescently (Cy3) labeled DNA nanostructures (with final concentration of 50 nM) in cell culture medium (FBS free) for 12 h. Before examination, the medium was removed and the cells were washed with PBS three times. Fresh growth medium was added, and the live cells were visualized by laser confocal fluorescent microscopy (Leica TCS SP5, German). The excitation wavelength of Cy3-labeled DNA nanostructures was 561 nm, and the corresponding emission filter was 565–600 nm.

In order to assess the subcellular localization of DNA nanostructures, SYTO 59 (S11341, Invitrogen, USA) was used to stain the cell nuclei. After incubating with fluorescently labeled DNA nanostructures, cells were washed three times with PBS and SYTO 59 was added to the growth medium with a final concentration of 0.5 μM. Cells were then incubated for a further 15 min before being washed and examined.

### Endotytic mechanism analysis

A549 cells (~5 × 10^4^) were seeded in 24-well plates (for fluorescence-activated cell sorting, FACS) or four-chamber glass bottom dish (for confocal imaging) and grown overnight prior to studies. They were pretreated with 500 μL of 1640 medium (FBS free) that contained different concentrations of chemical inhibitors for different period of time (see details in Supplementary Table 5). Then, cells were washed with PBS for three times and treated with 50 nM DNA nanostructures containing the same concentrations of chemical inhibitors for another 6 h. Following incubation, FACS and confocal analysis were performed. DNA nanostructure-treated-only cells were utilized as positive control and their fluorescence intensity was defined as 100%.

### Structural conformation and mechanical compliance simulation

The structural shape and mechanical compliance of DHT nanofilaments are modeled using the finite element model, which is developed as program CanDo (cando-dna-origami.org), to predicate the structural shape and mechanical flexibility of DNA structures. DNA nanofilaments are modeled as homogeneous elastic rods with isotropic bending stiffness. The structural and mechanical parameters of two-node beam finite elements composing each rod were listed as below.

B-form DNA helix is modeled as worm-like chain.

Axial length per base pair: 0.34 nm;

Helical diameter: 2.25 nm;

Basepairs per turn: 10.5;

Bending stiffness: 230 pN nm^2^;

Stretching modulus: 1100 pN;

Torsional stiffness: 460 pN nm^2^.

The backbone bending and torsional stiffness are reduced by 100-fold when there are nicks in the DNA double helix and single-stranded DNA used as sticky ends or loops are modeled as entropic springs using modified freely jointed chain model. Interhelical crossovers are treated as rigid component with zero length.

The structural conformation and mechanical complicance of DHT nanofilaments at ground-state solution are performed by normal mode analysis^61,62^ with CanDo. The thermal fluctuations are quantified using computing RMSFs of basepairs within nanofilaments are computed at 298 K. Here, RMSF measures the magnitude of motion of basepairs and exhibit flexibility of DNA nanofilaments.

### Reporting summary

Further information on experimental design is available in the Nature Research Reporting Summary linked to this article.

## Supplementary information

Supplementary Information

Reporting Summary

## Data Availability

The data that support the findings of this study are available within the paper and its Supplementary Information and are available from the corresponding authors upon reasonable request.

## Source data

Source Data
