# Supplementary material for: Programming chain-growth copolymerization of DNA hairpin tiles for in-vitro hierarchical supramolecular organization
Source: Nat Commun. 2019 Mar 1;10:1006. doi: 10.1038/s41467-019-09004-4 (PMC6397255; doi:10.1038/s41467-019-09004-4)
Supplement: Supplementary file 1 — Supplementary Information [file 41467_2019_9004_MOESM1_ESM.pdf]

Supplementary Information for

Programming chain-growth copolymerization of DNA hairpin tiles for in-vitro hierarchical supramolecular organization

Zhang, Wang, Zhang & Liu et al.

This PDF file includes:

Supplementary Figures 1 to 19

Supplementary Tables 1 to 5

Supplementary Notes 1 to 6

Supplementary References

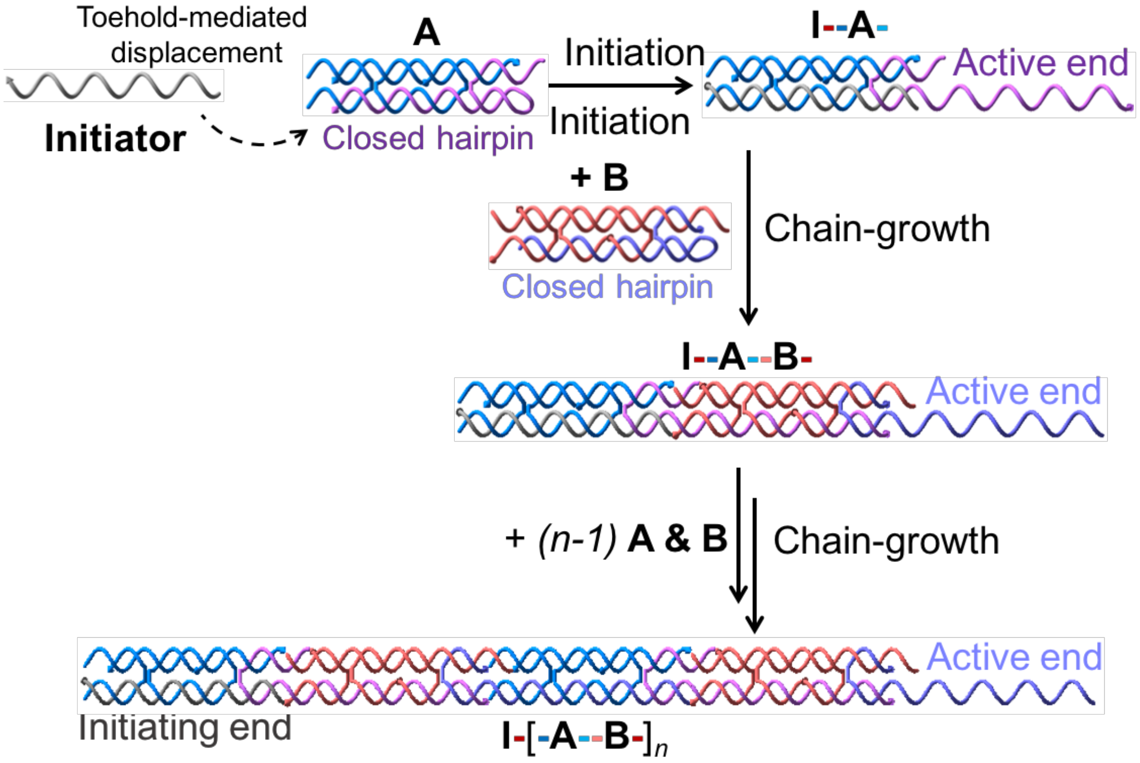

Supplementary Figure 1 | Structural schematics of individual DHT monomers, intermediates and copolymeric nanofilaments in one-dimensional chain-growth copolymerization process.

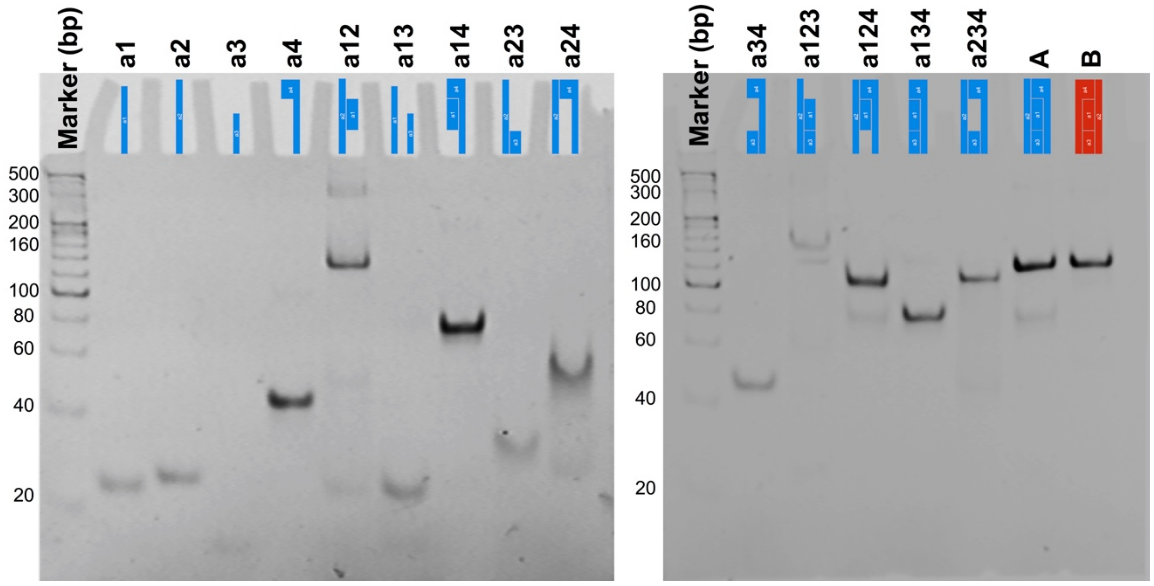

Supplementary Figure 2 | Native polyacrylamide gel electrophoresis (PAGE) of DNA sequences and assembled DHT monomers. Here the formation of monomers was verified by the PAGE analysis. Marker, 20 bp DNA Ladder. Lanes 1-4, single-stranded DNA a1 to a4 of monomer A; Lane 5, a1+a2; Lane 6, a1+a3; Lane 7, a1+a4; Lane 8, a2+a3; Lane 9，a2+a4; Lane 10, a3+a4; Lane 11, a1+a2+a3; Lane 12, a1+a2+a4; Lane 13, a1+a3+a4; Lane 14, a2+a3+a4; Lane 15, monomer A (a1+a2+a3+a4); Lane 16, monomer B (b1+b2+b3+b4).

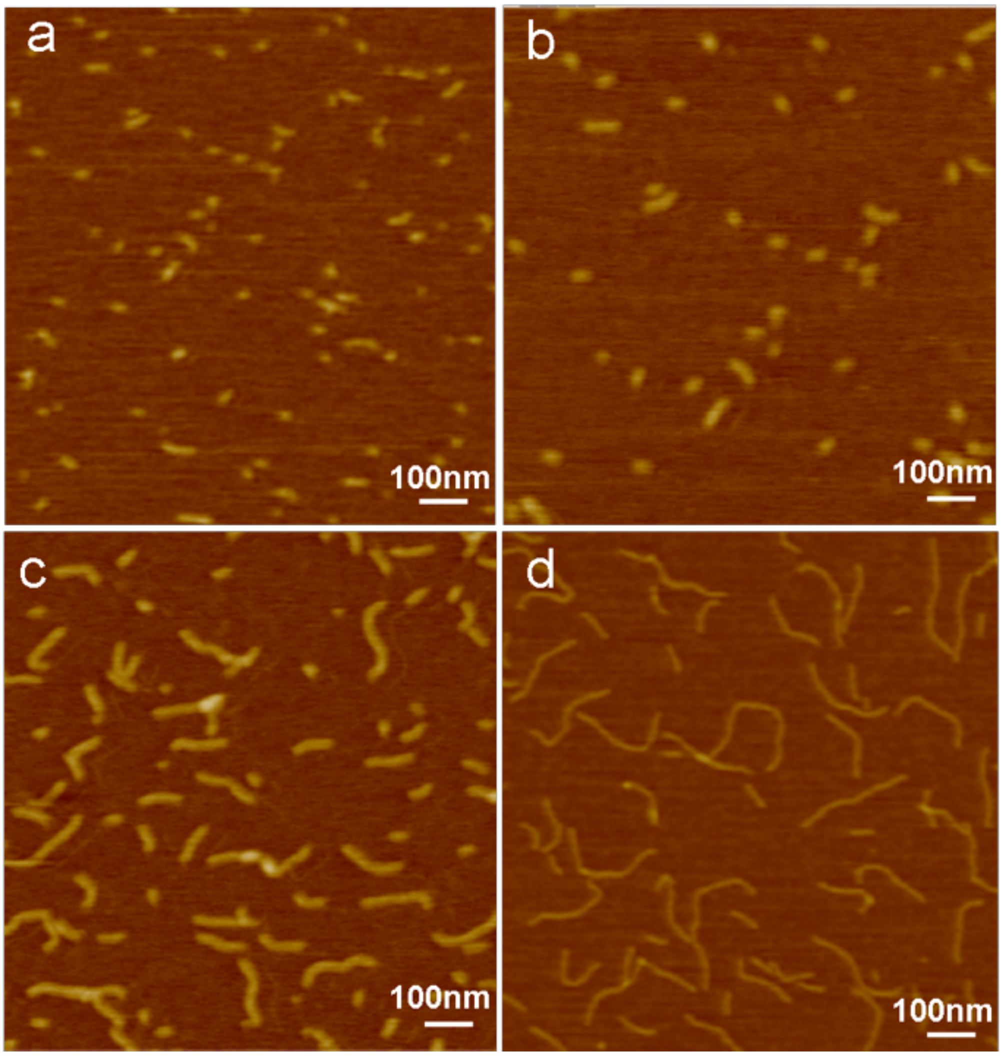

Supplementary Figure 3 | AFM images of copolymeric DHT nanofilaments at differernt monomer-to-initiator ratios ($[M]_0/[I]_0$). The concentration of monomers is kept constant ($[\mathbf{A}]_0$ = $[\mathbf{B}]_0$ = $[\mathbf{M}]_0$ = 1 μM). **a**, $[\mathbf{M}]_0/[\mathbf{I}]_0$ =1. **b**, $[\mathbf{M}]_0/[\mathbf{I}]_0$ =2. **c**, $[\mathbf{M}]_0/[\mathbf{I}]_0$ =5. **d**, $[\mathbf{M}]_0/[\mathbf{I}]_0$ =10.

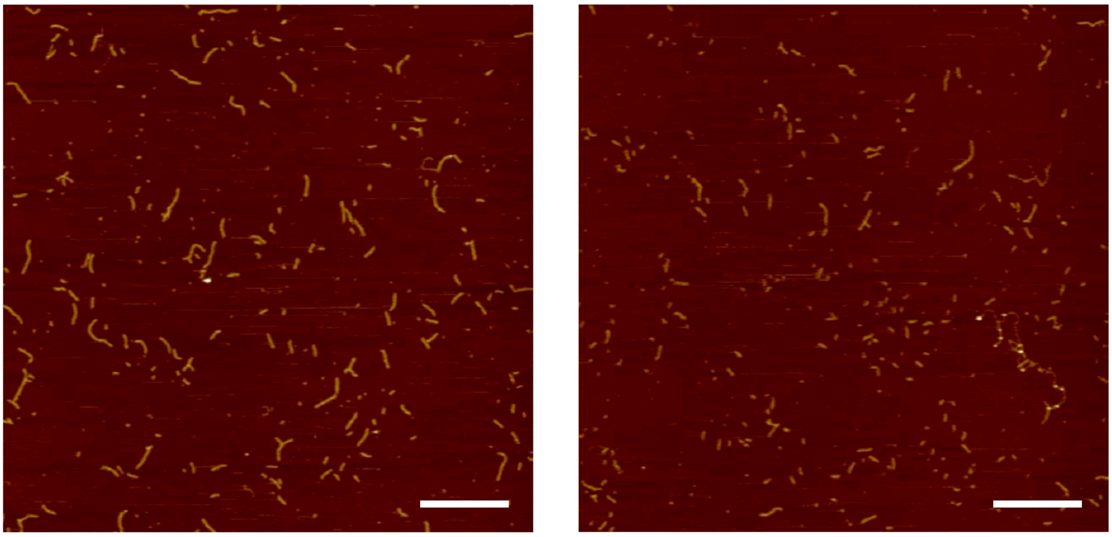

Supplementary Figure 4 | AFM images of products from thermal copolymerization of DHTs without initiator. 1 μM of monomers **A** and **B** at equimolar concentrations was mixed and heated at 45 ºC for 1 h and then incubated at room temperature for 30 min. The products from copolymerization of monomers without initiators were characterized with AFM. The sample exhibited high polydispersity with PDI of 1.82 (Fig. 2d inset, Ln= 192.0 nm, Lw= 348.6 nm,) . Scale bar: 500nm.

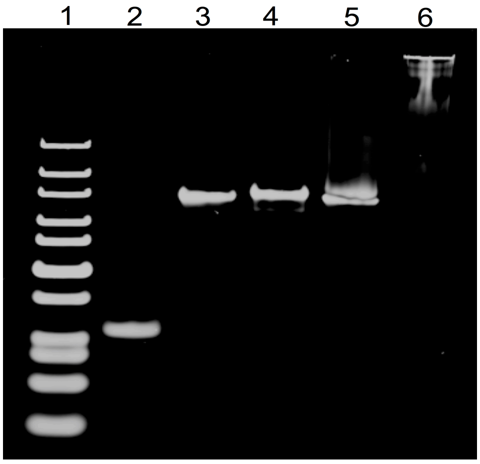

Supplementary Figure 5 | Native PAGE gel of monomers and products w/o and w/ initiator. Lane 1, marker (GeneRuler ultra low range DNA ladder, cat. #SM0371, ThermoFisher); Lane 2: initiator strand **I**; lane 3: monomer **A**; lane 4, monomer **B**; lane 5 and 6, the mixture of monomers **A** and **B** without (lane 5) and with (lane 6) initiator strand after incubation in room temperature. We employed PAGE to analyze initiator strand (lane 2), monomer **A** (lane 3), monomer **B** (lane 4), the mixture of monomers **A** and **B** without (lane 5) and with (lane 6) initiator strand. The mixture of monomers A and B w/o or w/ initiator were incubated for 1 h at room temperature and the ratio of monomers and initiator was 1:0.1 for lane 6. As shown in Fig. S3, we find that the band for the mixture of monomers **A** and **B** in the absence of initiator (lane 5) is in the same position as those for the monomer **A** (lane 3) and monomer **B** (lane 4) alone. In contrast, the presence of the initiator strand initiated the copolymerization of monomers, and the formed copolymers showed delayed bands in the lane 6. Thus, no copolymerization happened between monomer **A** and **B** without adding the initiator strand.

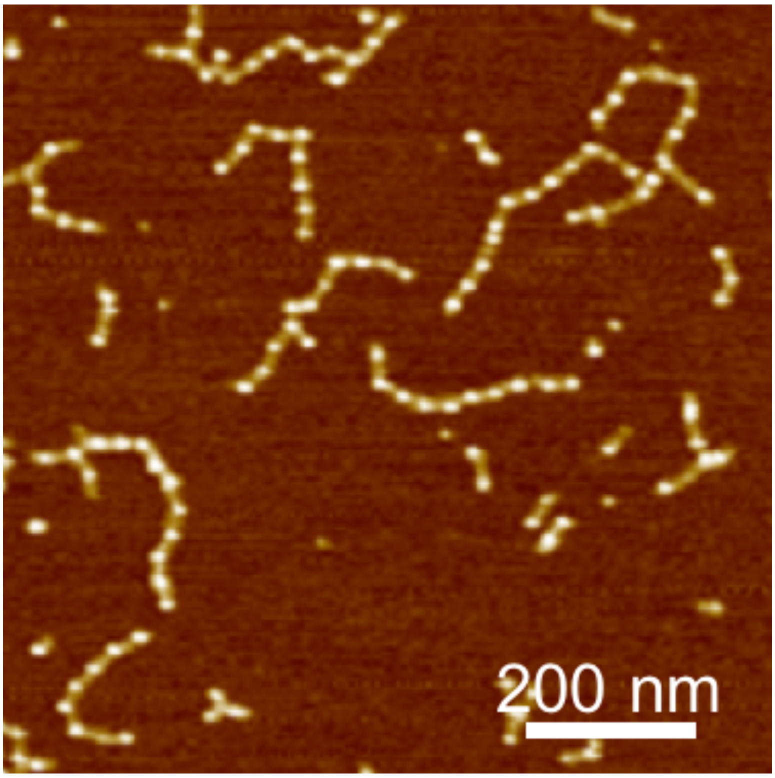

Supplementary Figure 6 | AFM images of STV-labeled DHT nanofilaments copolymerized at $[M]_0/[I]_0$ =10. Scale bar: 200nm.

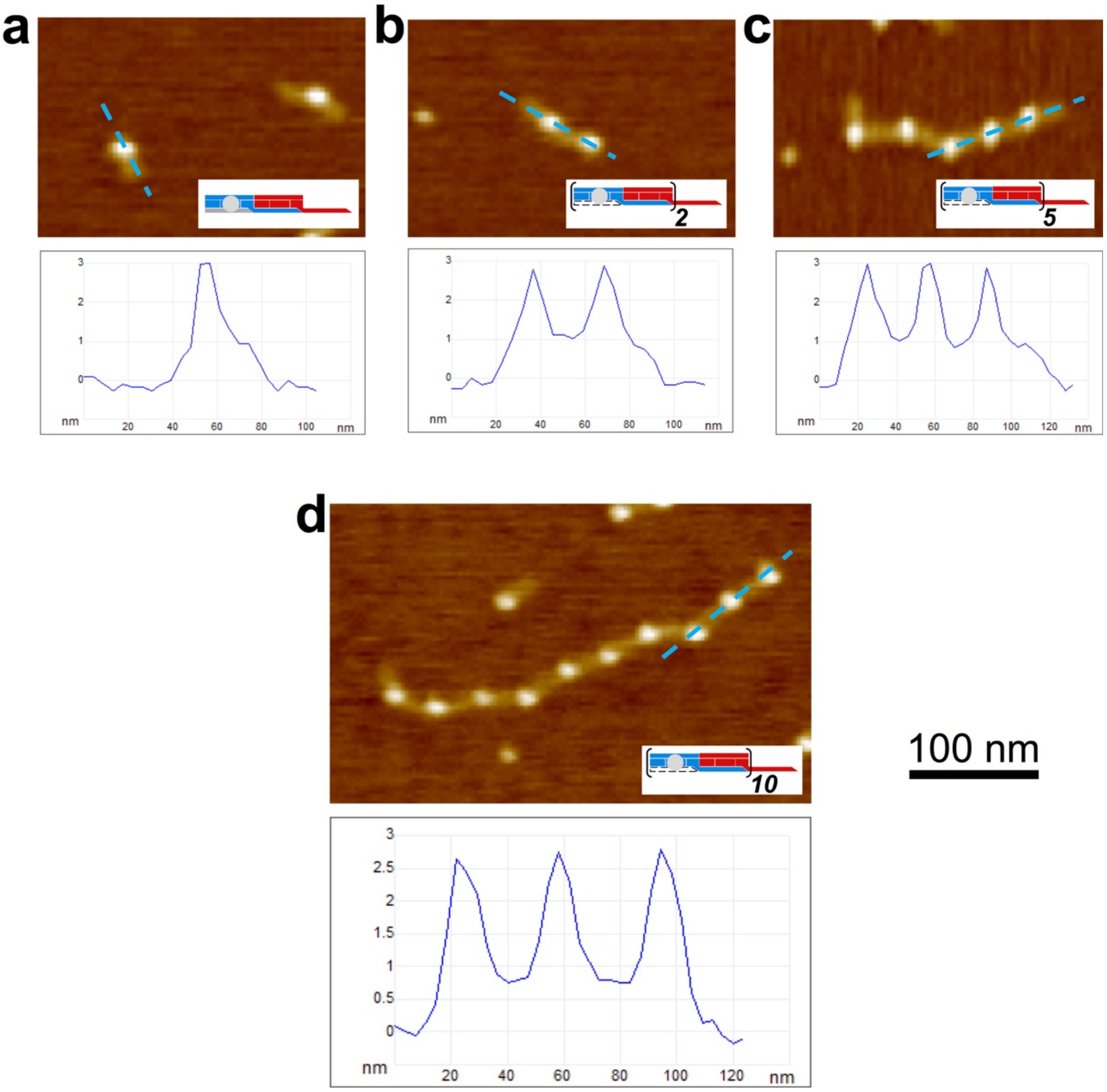

Supplementary Figure 7 | Height images of AFM micrographs and their cross-sectional profiles of polymerized mixtures of monomers A and B with I at (a) $[M]_0/[I]_0$ =1, (b) $[M]_0/[I]_0$ =2, (c) $[M]_0/[I]_0$ =5, (d) $[M]_0/[I]_0$ =10. The heights of STV-DHT nanofilaments are measured to about 3.1 nm and the heights of DHT nanofilaments are measured to about 1.2 nm.

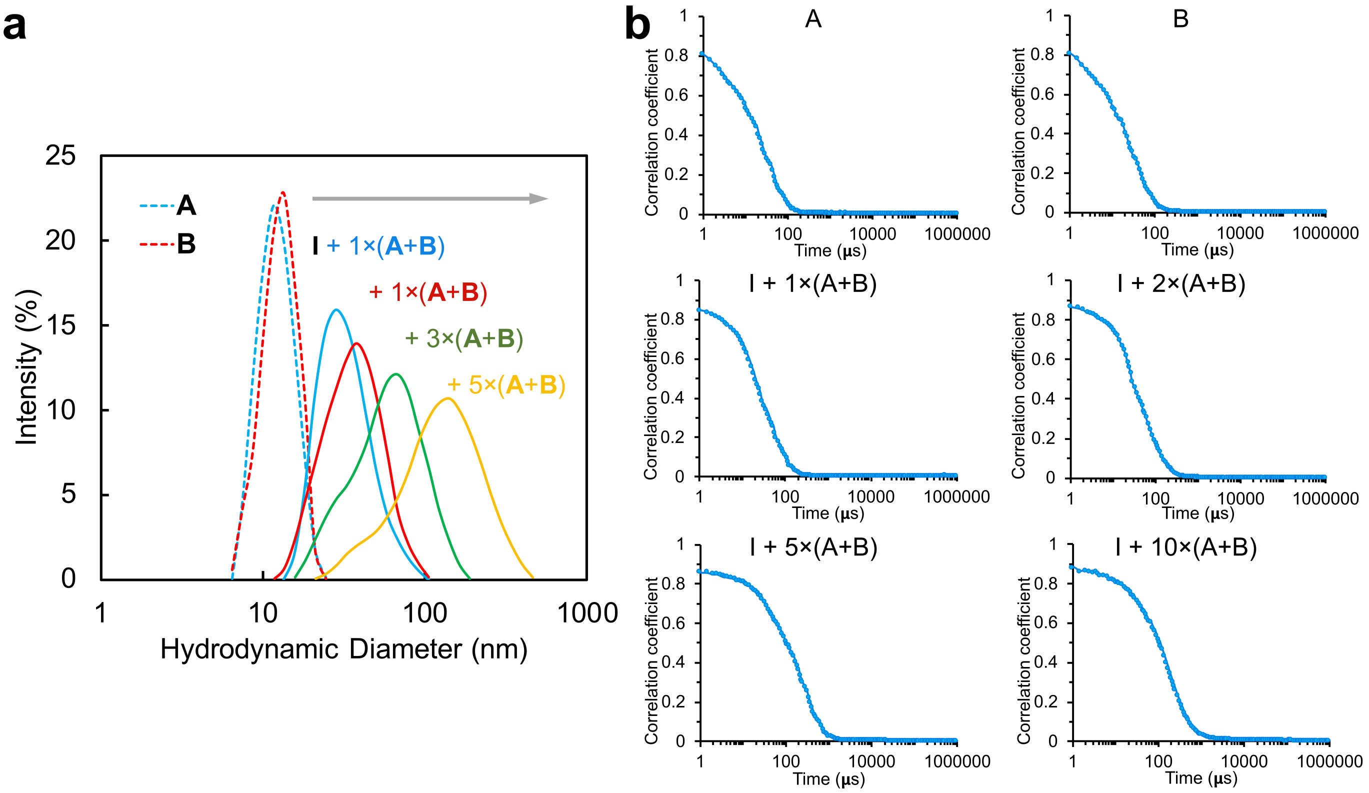

Supplementary Figure 8 | Dynamic light scattering (DLS) intensity distribution plots (a) and autocorrelation function curves (b) of living chain-growth copolymerization with the stepwise addition of monomers into initiator I. a, the DLS curve with blue dashed line corresponds to monomers **A** and the one with red dashed line to monomer **B**. The DLS curves with blue, red, green and yellow solid lines correspond to the DHT nanofilaments that copolymerized with finial [**M**]/[**I**]=1:1, 2:1, 5:1 and 10:1, respectively. The PDI values for different DNA nanostructures: 0.22 (A), 0.19 (B), 0.39 (1:1), 0.43 (2:1), 0.59 (5:1) and 0.72 (10:1).

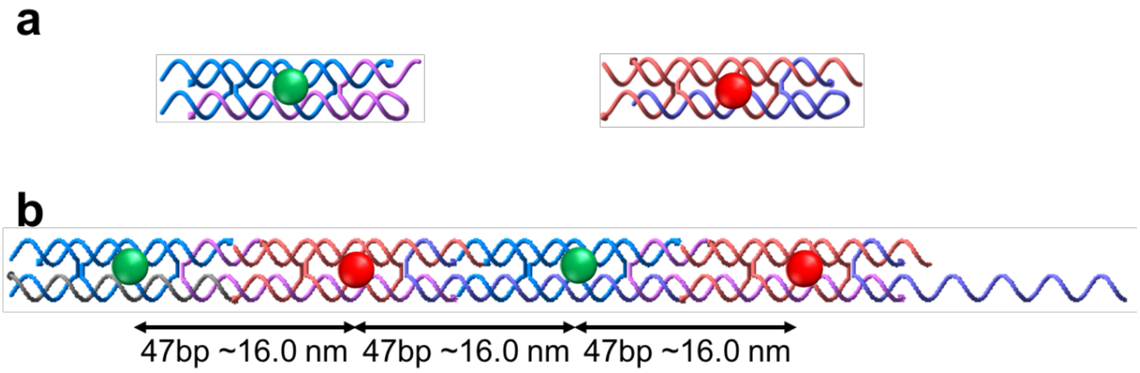

Supplementary Figure 9 | Schematic illustration of the distance between fluorescent dyes Cy3 and Cy5 labelled on DHT nanofilaments. Green and red dots correspond to dye Cy3 and Cy5, respectively. **a**, Cy3 was labeled on the 5' end of strand **a1** (left) and Cy5 was labelled on the 5' ends of **b1** (right). **b**, the positon of dyes Cy3 and Cy5 and inter-dye distance of the copolymerized DHT nanofilaments.

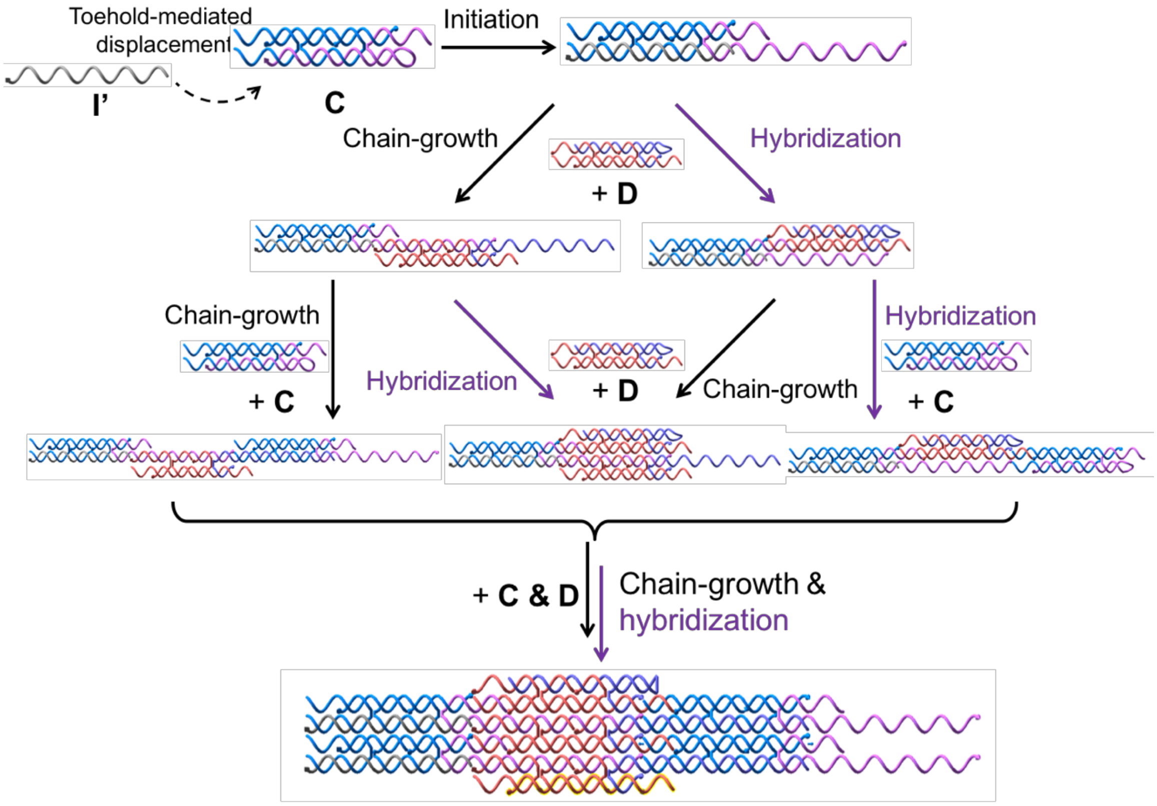

Supplementary Figure 10 | Structural schematics of individual DHT monomers, intermediates and copolymeric nanoplatelets in two-dimensional copolymerization process.

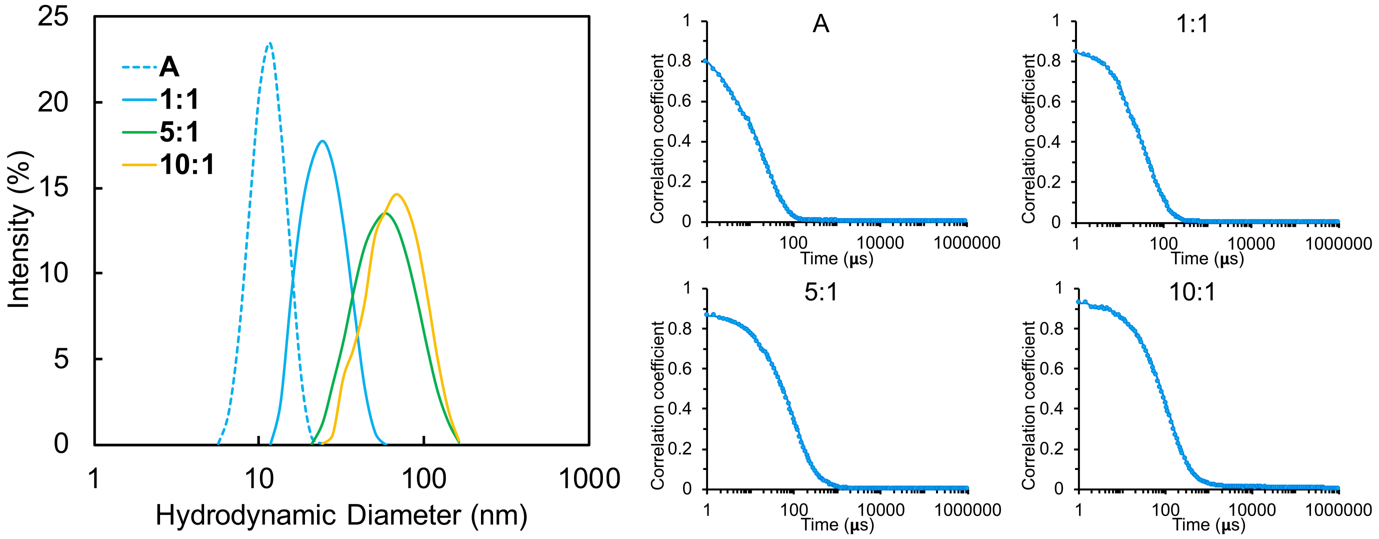

Supplementary Figure 11 | Dynamic light scattering (DLS) intensity distribution plots (a) and autocorrelation function curves (b) of monomer A and nanofilaments after incubation in the FBS-free 1640 cell culture medium. a, the DLS curve with blue dashed line corresponds to monomers **A**. The DLS curves with blue, green and yellow solid lines correspond to the DHT nanofilaments that copolymerized with finial [**M**]/[**I**]=1:1, 5:1 and 10:1, respectively. The PDI values for different structures: 0.23 (A), 0.39 (1:1), 0.37 (5:1) and 0.39 (10:1).

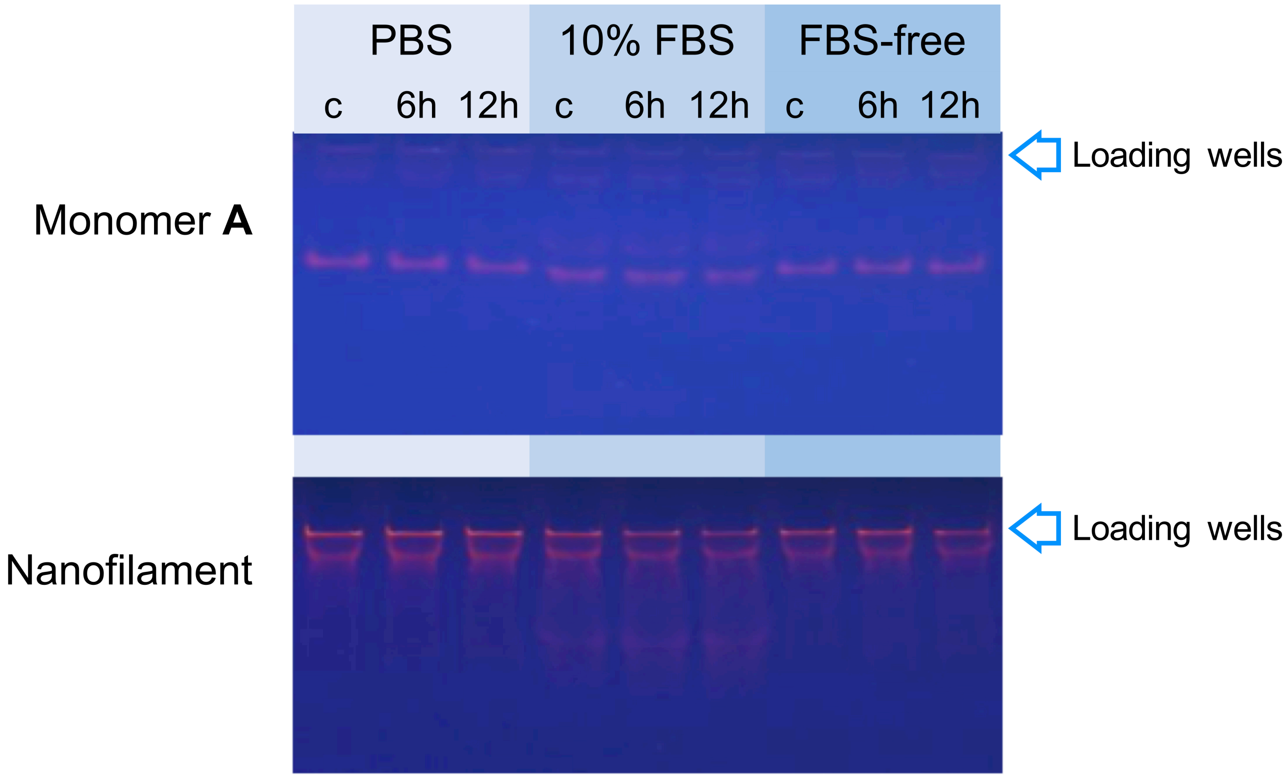

Supplementary Figure 12 | Native PAGE analysis of the stability of DHT nanofilaments in different circumstances. **A** and DHT nanofilaments (**I**-[**A**-**B**]$_{10}$) of the same DNA concentration (50 nM in means of DNA strands) were incubated with different solutions (PBS buffer, 10% FBS and FBS-free 1640 medium) and incubation time (6h and 12h). Label ‘c’ corresponds to control sample. We studied the extracellular stability of the nanofilaments against degradation through native polyacrylamide gel electrophoresis (PAGE). Monomer Native PAGE showed that the bands of the nanofilament remained almost unchanged after 12 h incubation, indicating the presence of intact structures.

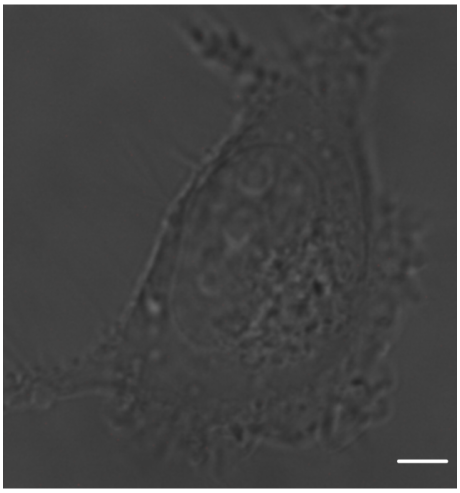

Supplementary Figure 13 | Confocal image of dsDNA after incubation with cells for 12 h. Cell lines: A549. Scale bars: 10 µm.

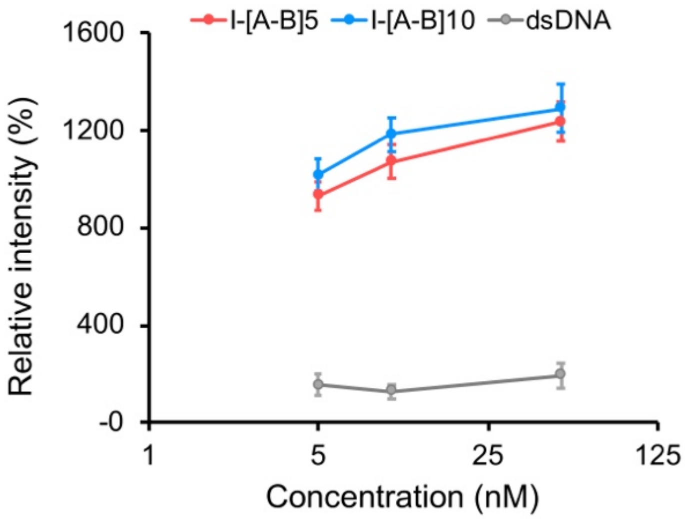

Supplementary Figure 14 | Fluorescence analysis of cellular uptake of DNA materials with different molar concentrations (5, 10 and 50 nM) in 12 h incubation. All error bars represent standard deviation obtained from at least three independent experiments.

The endocytosis experiment of DNA nanofilaments **I**-[**A**-**B**]$_5$, **I**-[**A**-**B**]$_{10}$ and dsDNA was carried out in molar concentrations ranging from 5, 10 to 50 nM with 12 h incubation . The results demonstrate that higher molar concentrations of nanofilaments lead to relative higher cellular uptake. The trend shown in Fig. 5c would not change even when considering the molar concentration effect.

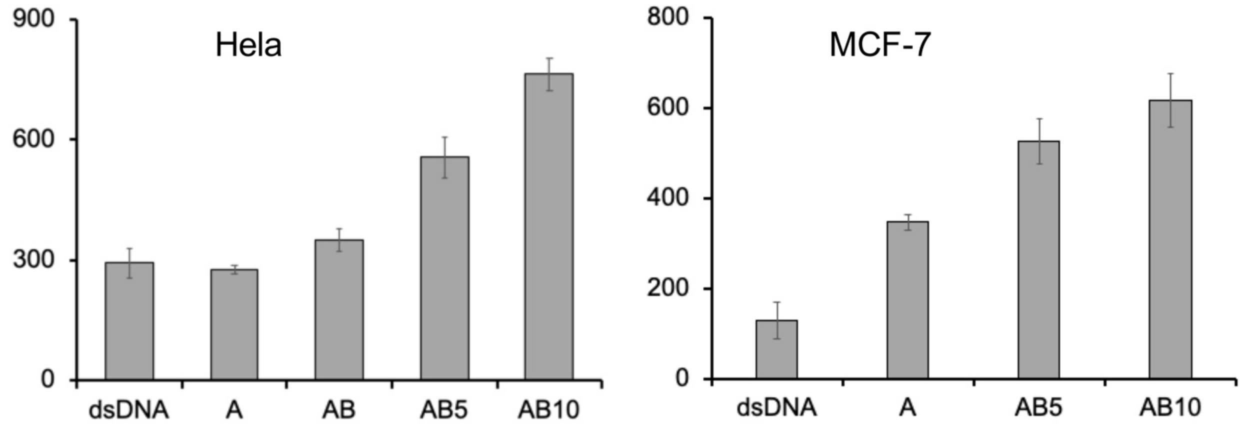

Supplementary Figure 15 | Fluorescence analysis of the dependence of cellular uptake on cell lines Hela (left) and MCF-7 (right). All error bars represent the standard deviation obtained from at least three independent experiments.

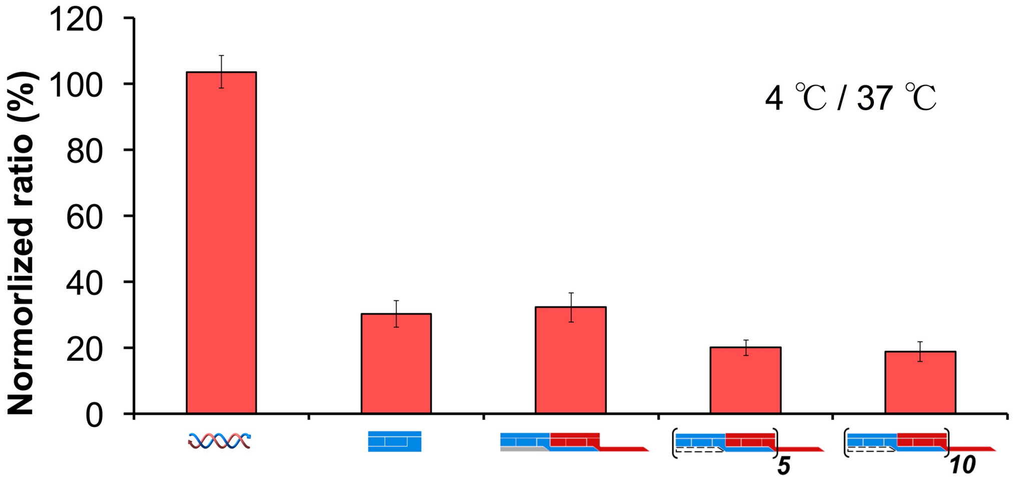

Supplementary Figure 16 | Fluorescence analysis of cellular uptake of DNA materials at different temperature (4 ºC/37 ºC), which were incubated with cells for 6 h. The normalized ratio stands for the fluorescence intensity measured at 37 ºC divided by that at 4 ºC. For dsDNA, 100% normalized ratio means that the fluorescence intensity is the same at 37 ºC and at 4 ºC, suggesting that the observed fluorescence for the dsDNA sample comes from surface-adsorbed dsDNA. For DNA nanostructures, incubation at 4 significantly reduced the fluorescence intensity, suggesting that the observed fluorescence at 37 ºC arises primarily from the cellular internalization. Cell lines: A549. All error bars represent the standard deviation obtained from at least three independent experiments.

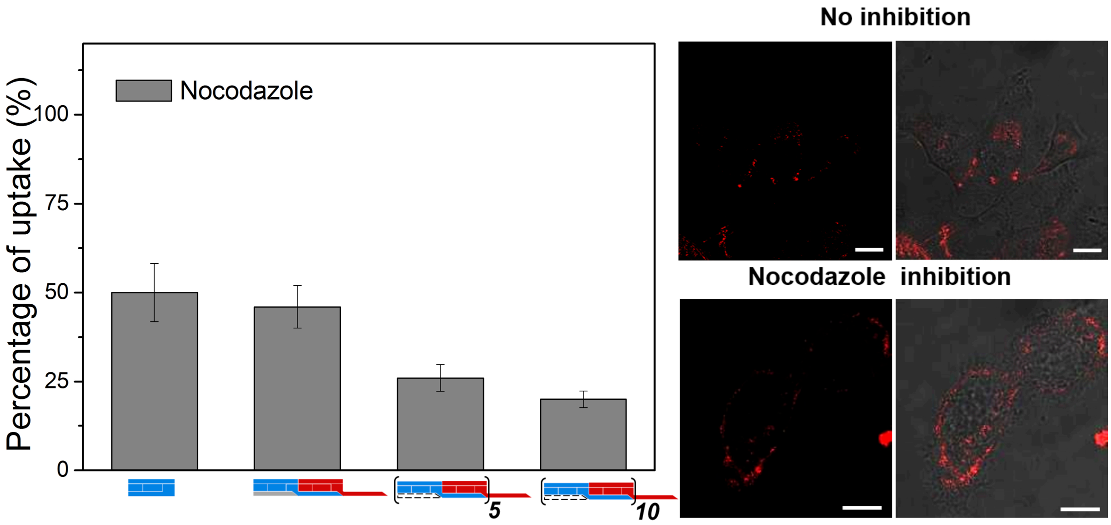

Supplementary Figure 17 | Flow cytometry (left) and confocal images (right) of nocodazole inhibition on cellular uptake of DHT nanofilaments. Cell lines: A549. All error bars represent the standard deviation obtained from at least three independent experiments. Scale bars: 10 μm.

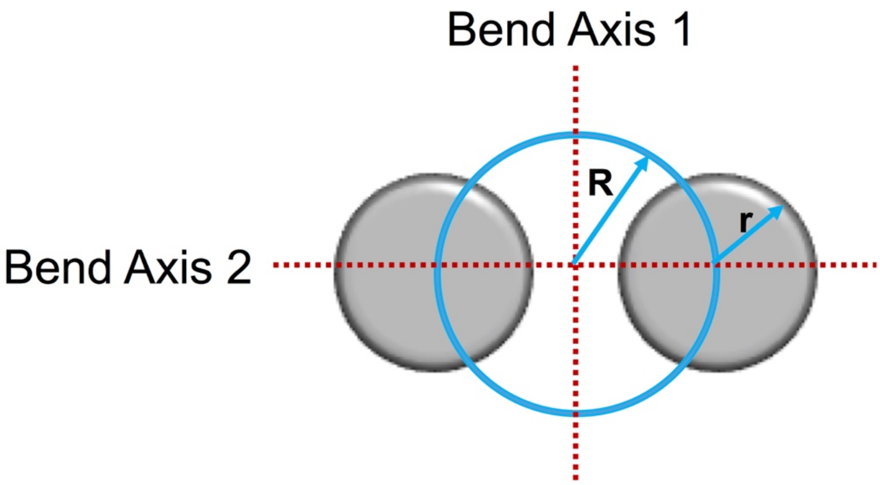

Supplementary Figure 18 | Calculation of the relative moment of inertia (*I*) for DHT nanofilament. Two red dotted lines indicate the perpendicular bend axes.

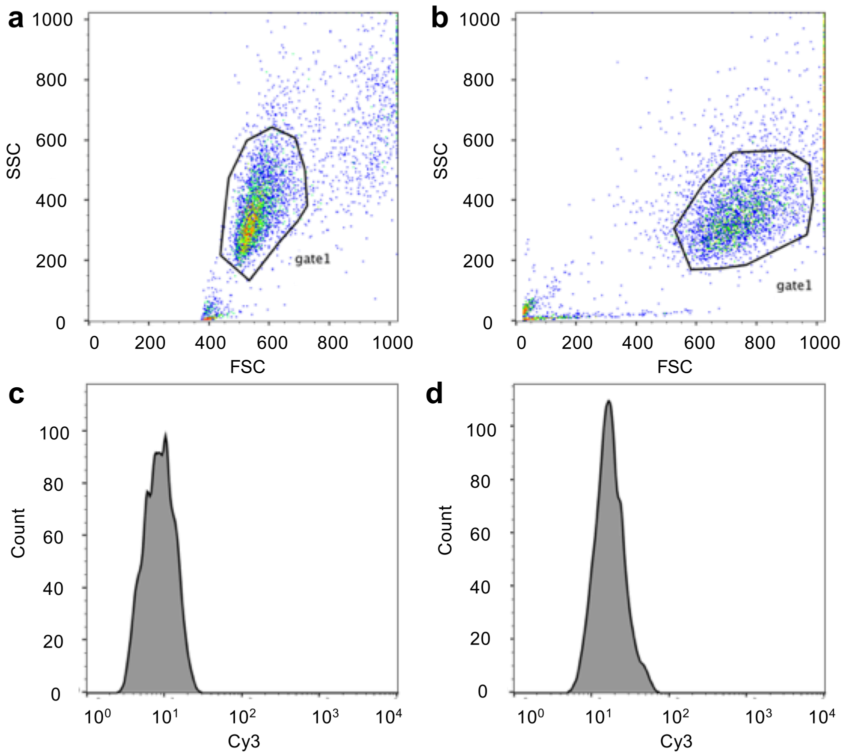

Supplementary Figure 19 | Gating strategy of flow cytometry experiments. a & **c**, control; **b** & **d**, inbubation of the Cy3-labeled **I-[A-B]**$_5$ with A549 cells for 6h. **a** & **b**, the x-axis represents the forward scatter (FSC) parameter which is relative to the size for the cell. The y-axis shows the side scatter (SSC) parameter which correlates with the components inside the cell. **c** & **d**, The x-axis represents the fluorophore intensity, while the y-axis indicates the cell counts in corresponding fluorescence intensity. We applied FSC and SSC to exclude cell debris and doublets by gating on the main cell population. Gate 1 indicates the main population of the cells we analyzed (**a**, 82.9%; **b**, 80.5%).

Supplementary Table 1 | Sequences of oligonucleotides for 1D DHT nanofilaments.

Name	Sequence (5'-3')
**a1**	GGACTTGTAGCGATACGACTCCGACGAGACTAGTAACTCTTG
**a2**	GGATGCGGAATGACAGCTACAAGTCCCAAGAGTTACGCTCTCCATTC
**a3**	TGTCACAGTAAGTCTTGTCATTCC
**a4**	GGGCTTGAATGGAGAGCCATCACTCATGTGAACCCATGAGTGATGTAGTCTCGTCGGAGTCG TATCAGACTTACT
**b1**	ACGAGACTACATGGTCAGATTCGTAGGTCCGATACGACTCCG
**b2**	GCATCCGATCCGTCCTGTCGGACCTACGAATCTGACCACCGAGAATC
**b3**	AAGCCCGATTCTCGGTCACTCATGGGTTCA
**b4**	CATGAGTGATGTAGTCTCGTCGGAGTCGTATCAGACTTACTGTGACAAGTAAGTCTGACAGG ACGGATC
**I**	CATGAGTGATGTAGTCTCGTCGGAGTCGTATCAGACTTACTGTGACA
**Bio-a1**	biotin-GGACTTGTAGCGATACGACTCCGACGAGACTAGTAACTCTTG
**Cy3-a1**	Cy3-GGACTTGTAGCGATACGACTCCGACGAGACTAGTAACTCTTG
**Cy5-b1**	Cy5- ACGAGACTACATGGTCAGATTCGTAGGTCCGATACGACTCCG

Supplementary Table 2 | The lengths and PDI of DHT nanofilaments from the AFM and SAXS data. Number-averaged length (L_n), weight-averaged length (L_w), standard deviation (σ) and polydispersity index (PDI) were calculated from the AFM data. Length (L_{SAXS}) were calculated from the SAXS data.

Sample		**A**	**I-A-B**	**I-[A-B]$_2$**	**I-[A-B]$_5$**	**I-[A-B]$_{10}$**
Theoretical (nm)		16	32	64	160	320
AFM	L_n (nm)	-	35.6	69.0	168.5	331.5
	L_w (nm)	-	43.2	83.7	209.6	416.9
	σ (nm)	-	8.2	16.0	41.8	84.6
	PDI	-	1.21	1.21	1.24	1.26
SAXS	L_{SAXS} (nm)	15.4	30.8	47.5	158.1	261.8
	σ (nm)	0.1	2.9	1.8	91.7	33.6

Supplementary Table 3 | Sequences of oligonucleotides for 2D DHT nanoplatelets.

Name	Sequence (5'-3')
**c1**	GGGACTTGTAGATACGACTCCGACGAGACTACCGTAACTCTT
**c2**	AAGTGGATGCCGAATGACAAGCTACAAGTCCCAAGAGTTACGCTCTCCATTC
**c3**	AGACTGTCACGAGTAAGTCTTCTTGTCATTCG
**c4**	GTAACGGGCTTGAATGGAGAGATCACTCATGCAGATGAACCGCATGAGTGATGTAGTCTCGT CGGAGTCGTATAAGACTTACTC
**d1**	GACGAGACTACTGGTCAGATTCGTAGGTCCGAATACGACTCC
**d2**	GCATCCACTTGATCCGTCCTGTCGGACCTACGAATCTGACCATCCGAGAATC
**d3**	AAGCCCGTCACGATTCTCGGAATCACTCATGCGGTTCATCTG
**d4**	CATGAGTGATGTAGTCTCGTCGGAGTCGTATAAGACTTACTCGTGACAGTCTGAGTAAGTCT TCAGGACGGATC
**l'**	CATGAGTGATGTAGTCTCGTCGGAGTCGTATAAGACTTACTCGTGACAGTCT

Supplementary Table 4 | The relative stiffness and compliance of DNA structures.

Structures	Width (nm)	Relative stiffness ($k_{structure}/k_{dsDNA}$)		Relative compliance ($k_{dsDNA}/k_{structure}$)	
		Theoretical	Experimental	Theoretical	Experimental
dsDNA (24 bp)	2	1		1	
Monomer **A**	5	0.83	0.90 ± 0.34	1.2	1.6 ± 0.4
**I-A-B**	5	0.10	0.10 ± 0.03	9.9	12.5 ± 2.6
**I-[A-B]**$_5$	5	8.1×10^{-4}	$(1.1 \pm 0.5) \times 10^{-3}$	1230.8	1755.9 ± 580.9
**I-[A-B]**$_{10}$	5	1.0×10^{-4}	$(2.8 \pm 1.1) \times 10^{-4}$	9846.2	13427.4 ± 5469.1

Supplementary Table 5 | List of chemical inhibitors of different pathways.

Target pathway	Chemical inhibitor	Final concentration	Preincubation time
Phagocytosis	Cytochalasin D	10 μg/mL	50 min
	Nocodazole	60 μM	50 min
Clathrin	Dansylcadaverine	100 μM	60 min
Caveolin	Genistein	200 μM	60 min

Supplementary Note 1 | Forster resonance energy transfer (FRET) pairs position design.

In single-molecule fluorescence imaging experiment, we designed the positions of fluorescent dyes Cy3 and Cy5, and inter-dye distance in the copolymeric one-dimensional nanofilaments to minimize the occurrence of FRET between dyes. The efficiency of FRET is extremely sensitive to the separation distance between dyes. The range over which the energy transfer can take place is limited to approximately 10 nm. As shown in Supplementary Figure 9, fluorescent dyes Cy3 and Cy5 were labeled on the centres of monomers **A** and **B** through binding to the 5' ends of **a1** and **b1**, respectively. In the copolymerized nanofilaments, the rigid strands between labeled dyes Cy3 and Cy5 is about 16.0 nm (47 bp) in length, therefore the FRET efficiency between Cy3 and Cy5 could not take place.

Supplementary Note 2 | Fluorescence analysis of the dependence of cellular uptake on cell lines Hela and MCF-7.

To check if there is any cell-type effect for our DNA structures, here we provide the internalization data of other two cell lines (Hela and MCF-7) incubated with DNA samples for 12 h (see Supplementary Figure 15). Five DNA structures (dsDNA, monomer A, nanofilaments **I**-**A**-**B**, **I**-[**A**-**B**]$_5$ and **I**-[**A**-**B**]$_{10}$) and four structures (dsDNA monomer A, nanofilaments **I**-[**A**-**B**]$_5$ and **I**-[**A**-**B**]$_{10}$) were used for endocytosis in Hela and MCF-7 cell lines, respectively. Although the internalization by Hela or MCF-7 was not so efficient as A549 cells, these results illustrated the similar increasing trends of uptake efficiency along with the shape and compliance of DNA structures. The results support our conclusion that the endocytosis of DNA nanofilaments we synthesized is structural and mechanical stiffness dependent, and no significant cell type effect found in the three cell lines we used.

Supplementary Note 3 | Nocodazole inhibition on cellular uptake of DHT nanofilaments.

Nocodazole, macropinocytosis inhibitor, was employed to verify if the internalization of one dimensional DHT nanofilaments was through the macropinocytosis pathway. The effects of nocodazole inhibition on cellular uptake efficiency of nanofilaments under FBS free incubation condition measured by flow cytometry and confocal images showing the distribution of ten pairs (**I-**[**A-B**]$_{10}$) DHT nanofilaments in cells pretreated with nocodazole or not. As shown in Supplementary Figure 17, results showed that all assembled DNA nanostructures presented a remarkable decrease (50-81%) in cellular internalization. Distribution of ten pairs (**I-**[**A-B**]$_{10}$) DHT nanofilaments before (in the plasma) and after (on the surface) nocodazole treatment was recorded by confocal microscopy, and results showed that nocodazole can significantly inhibit the internalization of the nanofilament.

Supplementary Note 4 | Calculation of the relative moment of inertia (*I*) for DHT nanofilament.

As shown in Supplementary Figure 18, we assume DHT nanofilament as a bundle of two rigidly linked DNA helices of radius *r* and no gaps between the helices. The moment of inertia of each helix with respect to its own center of mass is *i*, is displaced from the nanofilament's center of mass by a distance *R.* The Young's modulus of the nanofilament is the same as that of dsDNA. *R* = 1.5 nm, *r* = 1 nm.

Bend axis 1:

$$I/i = 2 \times [1+2 \times (1.5/1)^2] = 11 \qquad (1)$$

Bend axis 2:

$$I/i = 2 \times (1/1)2 = 2 \qquad (2)$$

Supplementary Note 5 | Calculation of the relative compliance of DNA structures.

The mechanical compliance of DNA structures is the inverse of stiffness. The bending stiffness (k_b) of beam-shaped structure:

$$k_b = \frac{3EI}{L^3} \qquad (3)$$

where *E* is the Young's modulus (elastic modulus),

L is the length of the DNA nanostructure (calculated from theoretical values or AFM experimental values as

shown in Table 2, L_n),

I is the area moment of inertia.

To estimate the moment of inertia, we treat a nanofilament as a bundle of N rigidly linked cylindrical rods of radius r, I could be calculated in terms of i[1, 2]. The moment of inertia of each dsDNA helix with respect to its own center of mass is i, is displaced from the nanofiament's center of mass by a distance R (Supplementary Figure 18 and Note 4). By the parallel axis theorem, the moment of inertia of the dsDNA helix with respect to the nanofiament's center of mass is $i+MR^2$, where M is the mass of the helix. Assuming uniform density,

$$i = \frac{1}{2}Mr^2 \qquad (4)$$

and thus

$$I = N(i + MR^2) = N\left(i + 2i\frac{R^2}{r^2}\right) \qquad (5)$$

Relative compliance

$$\frac{k_b(dsDNA)}{k_b(nanofilament)} = \frac{i}{I}\times\left(\frac{L(nanofilament)}{L(dsDNA)}\right)^3 = \frac{1}{N\left(1+2\frac{R^2}{r^2}\right)}\times\left(\frac{L(nanofilament)}{L(dsDNA)}\right)^3 \qquad (6)$$

Supplemenatry Note 6 | Chemical inhibitors of different pathways.

As shown in Supplementary Table 5, Cytochalasin D, which disrupts F-actin filaments *via* actin depolymerization, was used to identify whether phagocytosis or macropinocytosis pathway was included in the endocytosis process. Dansylcadaverine, which blocks the formation of coated pits by inhibiting transglutaminase in the cell membrane, was used to block clathrin-mediated endocytosis. Genistein, a tyrosine kinase inhibitor that inhibits actin recruitment in caveolae, was used to discern the role of caveolae-mediated endcytosis in internalization.

Supplementary References:

1. Rothemund, P.W. et al. Design and characterization of programmable DNA nanotubes. *J Am Chem Soc* **126**, 16344-16352 (2004).
2. Wang, T., Schiffels, D., Martinez Cuesta, S., Kuchnir Fygenson, D. & Seeman, N.C. Design and Characterization of 1D Nanotubes and 2D Periodic Arrays Self-Assembled from DNA Multi-Helix Bundles. *Journal of the American Chemical Society* **134**, 1606-1616 (2012).
